# Correction: Generation of Human Induced Pluripotent Stem (iPS) Cells in Serum- and Feeder-Free Defined Culture and TGF-β1 Regulation of Pluripotency

**DOI:** 10.1371/journal.pone.0093242

**Published:** 2014-03-18

**Authors:** The PLOS ONE Staff

## Notice of Republication

This article was republished on February 28, 2014, to correct capitalization in the title. The originally published, uncorrected article and the republished, corrected article are provided here for reference.

## Supporting Information

File S1 Originally published, uncorrected article.

File S2 Republished, corrected article.

## References

[1] Yamasaki S, Taguchi Y, Shimamoto A, Mukasa H, Tahara H, et al (2014) Generation of Human Induced Pluripotent Stem (iPS) Cells in Serum- and Feeder-Free Defined Culture and TGF-β1 Regulation of Pluripotency. PLoS ONE 9(1): e87151 doi:10.1371/journal.pone.0087151 24489856 10.1371/journal.pone.0087151 PMC3906124

