# Supplementary figures and images for: Generation of Human Induced Pluripotent Stem (iPS) Cells in Serum- and Feeder-Free Defined Culture and TGF-β1 Regulation of Pluripotency
Source: PLoS One. 2014 Jan 29;9(1):e87151. doi: 10.1371/journal.pone.0087151 (PMC3906124; doi:10.1371/journal.pone.0087151)

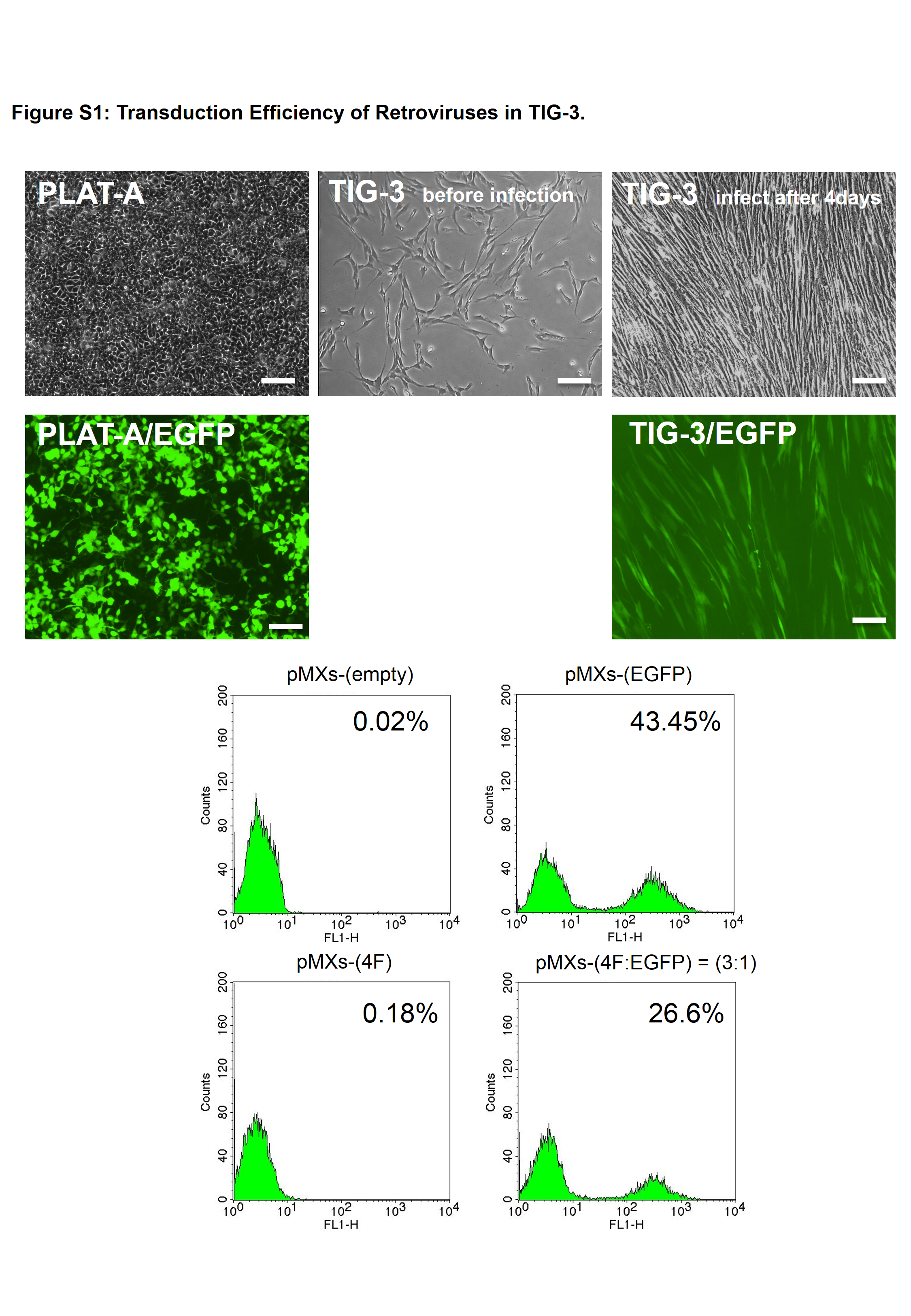

Supplement: Figure S1 — Transduction Efficiency of Retroviruses in TIG-3. TIG-3 was introduced with pMXs retroviruses containing the EGFP cDNA. After 4 days, cells were photographed under a fluorescence microscope and analyzed by flow cytometry. The upper panel shows the images of phase contrast and fluorescent microscope. The lower panel shows the result of flow cytometry. Shown are percentages of cells expressing EGFP. (TIF) [file pone.0087151.s001.tif]

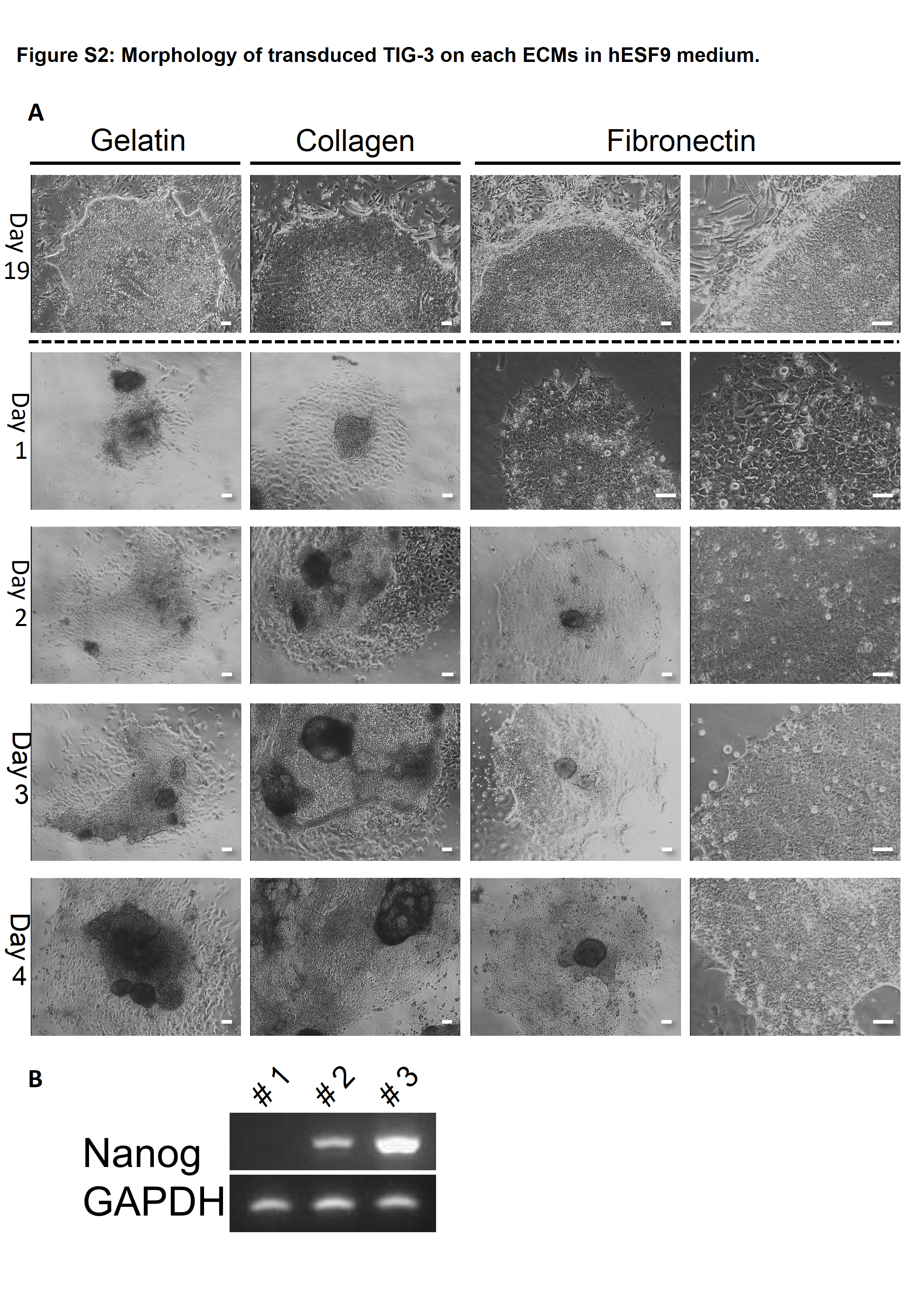

Supplement: Figure S2 — Morphology of transduced TIG-3 on each ECMs in hESF9 medium. A) Upper figures: Twenty days after transduction TIG-3-derived human iPS colony were picked up and sub-cultured on each ECMs. Lower figures: Images of sub-cultured iPS colonies seeded on each ECMs with hESF9 medium for the indicated days at the left. B) Expression of ES cell marker genes in iPSCs derived from TIG-3 cultured on each ECMs with hESF9 medium at day 4. The expression of pluripotency marker genes; Nanog were weakened or disappeared when picked up and sub-cultured on collagen and gelatin. We used primers that only amplified the endogenous genes. #1: hiPSCs generated from TIG-3 on gelatin-coated dish and sub-cultured on gelatin-coated dishes with hESF9 medium at passage 2. #2: hiPSCs generated from TIG-3 on collagen-coated dish and sub-cultured on collagen-coated dishes with hESF9 medium at passage 2. #3: hiPSCs generated from TIG-3 on fibronectin-coated dish and sub-cultured on fibronectin-coated dishes with hESF9 medium at passage 2. Bars indicate 200 µm. (TIF) [file pone.0087151.s002.tif]

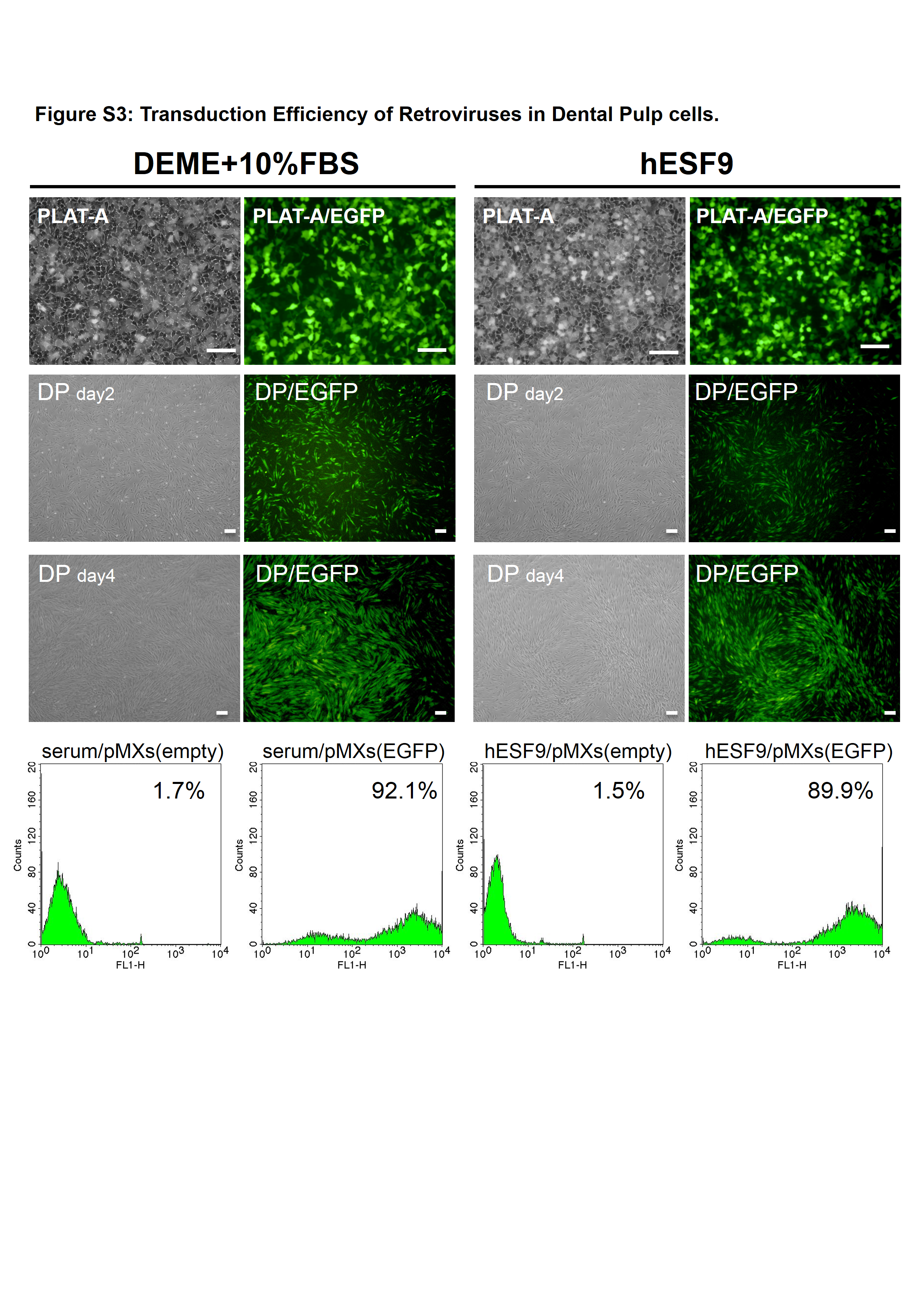

Supplement: Figure S3 — Transduction Efficiency of Retroviruses in Dental Pulp cells. DPCs were introduced with pMXs retroviruses containing the EGFP cDNA. After 4 days, cells were photographed under a fluorescence microscope and analyzed by flow cytometry. The upper panel shows the images of phase contrast and fluorescent microscope. The lower panel shows the result of flow cytometry. Shown are percentages of cells expressing GFP. Transfection efficiency of EGFP was 92.1% in serum-supplemented condition and 89.9% in serum-free culture condition of transfected cells. Bars indicate 200 µm. (TIF) [file pone.0087151.s003.tif]

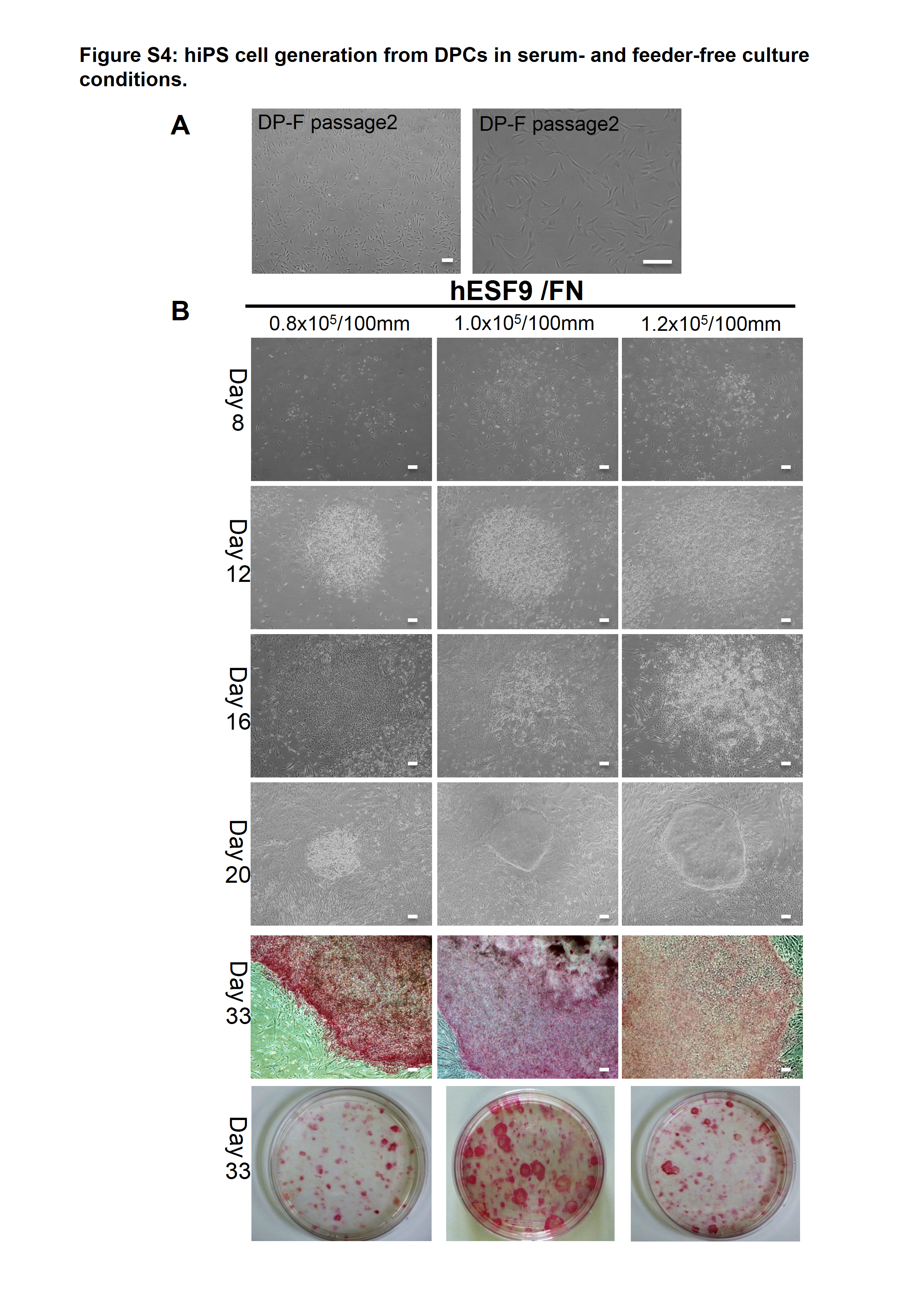

Supplement: Figure S4 — hiPS cell generation from DPCs in serum- and feeder-free culture conditions. Images of DPCs (DP-F) plated on collagen-coated dish in RD6F medium. A) Images of DPCs (passage 2) on type I collagen-coated plate with RD6F medium. B) Transduced DPCs were cultured on fibronectin with hESF9 medium or on MEF with KSR-based conditions. After 20 days, iPS colony were picked up and sub-cultured on fibronectin. The reprogramming efficiency was 0.25% with a high success rate. C) ALP staining of iPSCs on fibronectin at 33 days after infection. Bars indicate 200 µm. (TIF) [file pone.0087151.s004.tif]

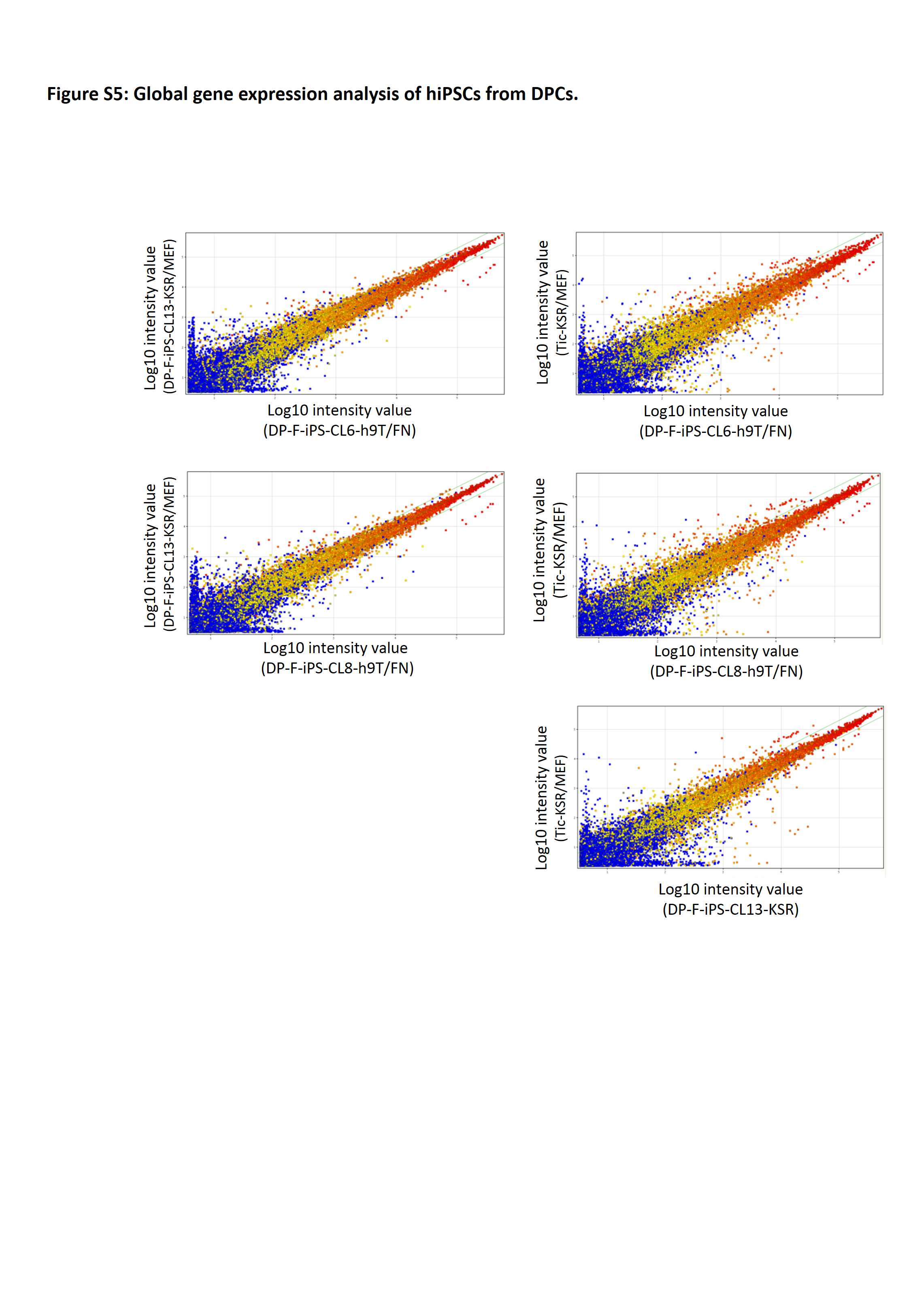

Supplement: Figure S5 — Global gene expression analysis of hiPSCs from DPCs. The gene expression of DP-hiPSCs generated in hESF9 and maintained in hESF9T is similar to that of the cells generated and maintained in conventional KSR-based condition or that of Tic (JCRB1331) maintained in conventional KSR-based condition. (TIF) [file pone.0087151.s005.tif]

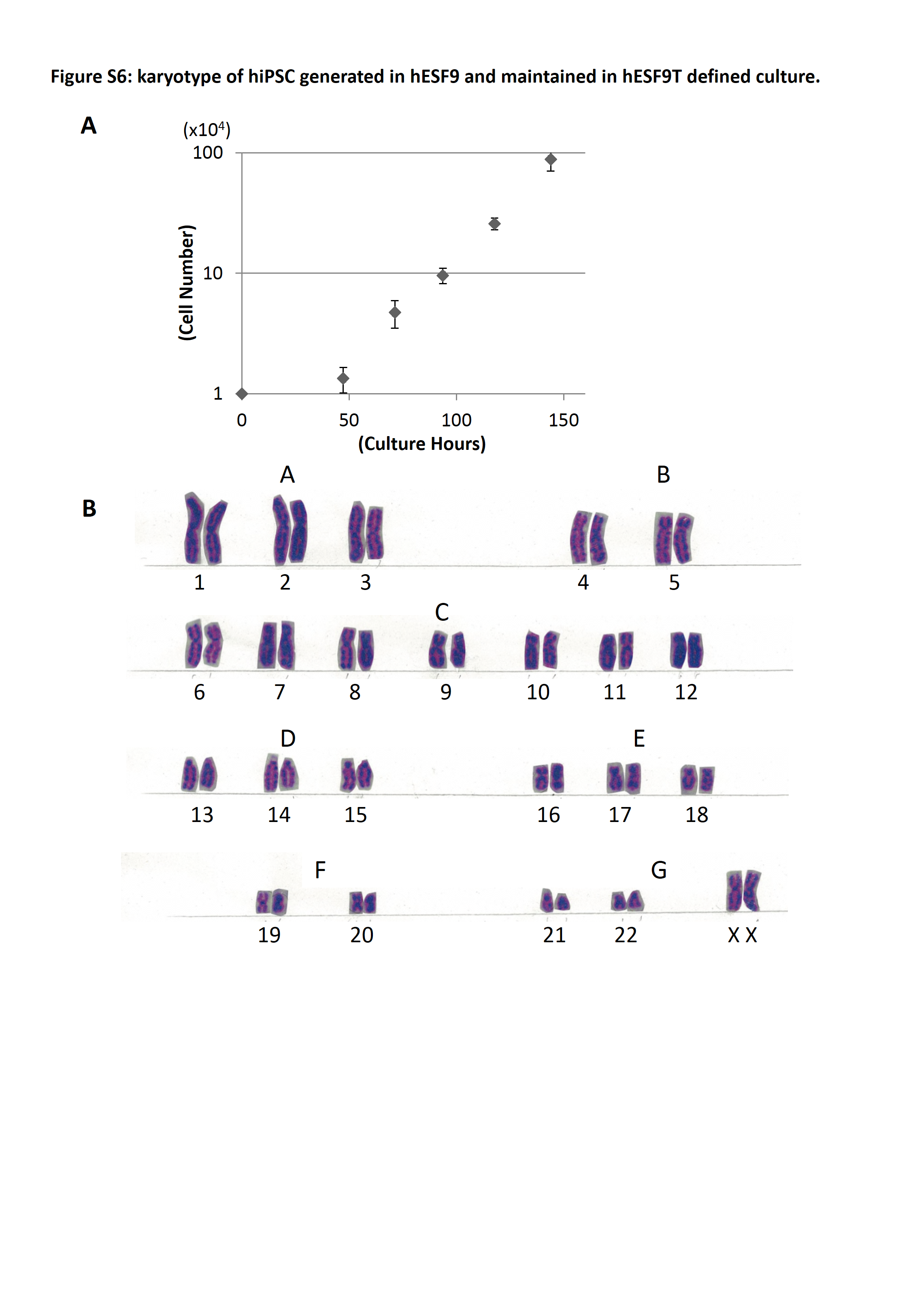

Supplement: Figure S6 — karyotype of hiPSC generated in hESF9 and maintained in hESF9T defined culture. A) Growth curve of hiPSCs. Shown were averages. Growth curves for the hiPSC (DP-F-iPS-CL16) cultured under hESF9T at passage 21, 22, 23 and 24 were seeded in a 24-well plate coated with fibronectin and the cell numbers were counted every 24 h. The values are the mean±SEM (n = 4). Population doubling time: 16.6±0.843 h. B) Karyotype analysis of DP-F-iPS-CL14 cell at passage 20 maintained in hESF9T conditions. Normal diploid 46, XX karyotype. (TIF) [file pone.0087151.s006.tif]

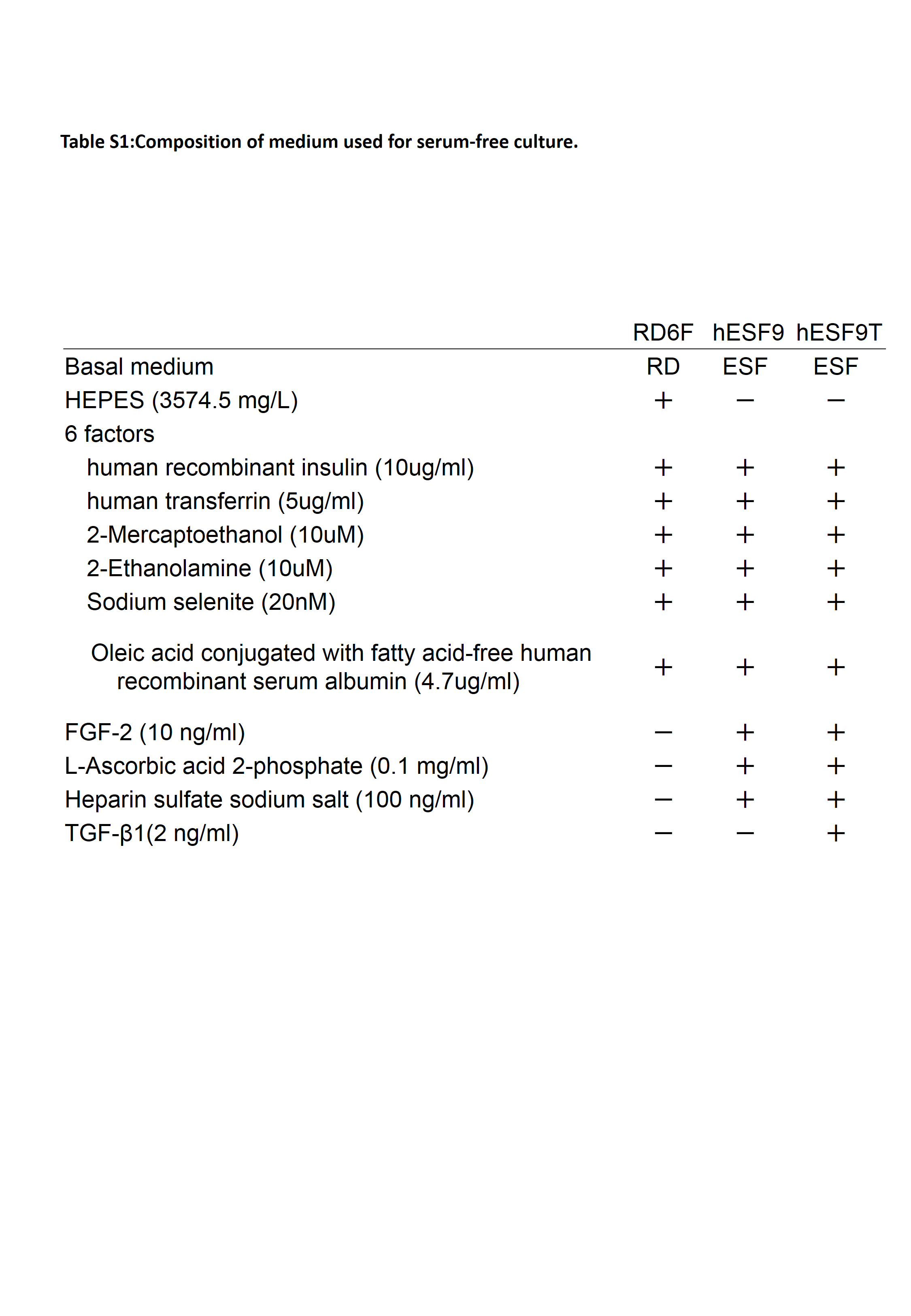

Supplement: Table S1 — Composition of medium used for serum-free culture. The composition of the basal medium RD is described in Sato, JD et al., 1987[11]. hESF9 medium is described in Furue et al., 2008 [5]. (TIF) [file pone.0087151.s007.tif]

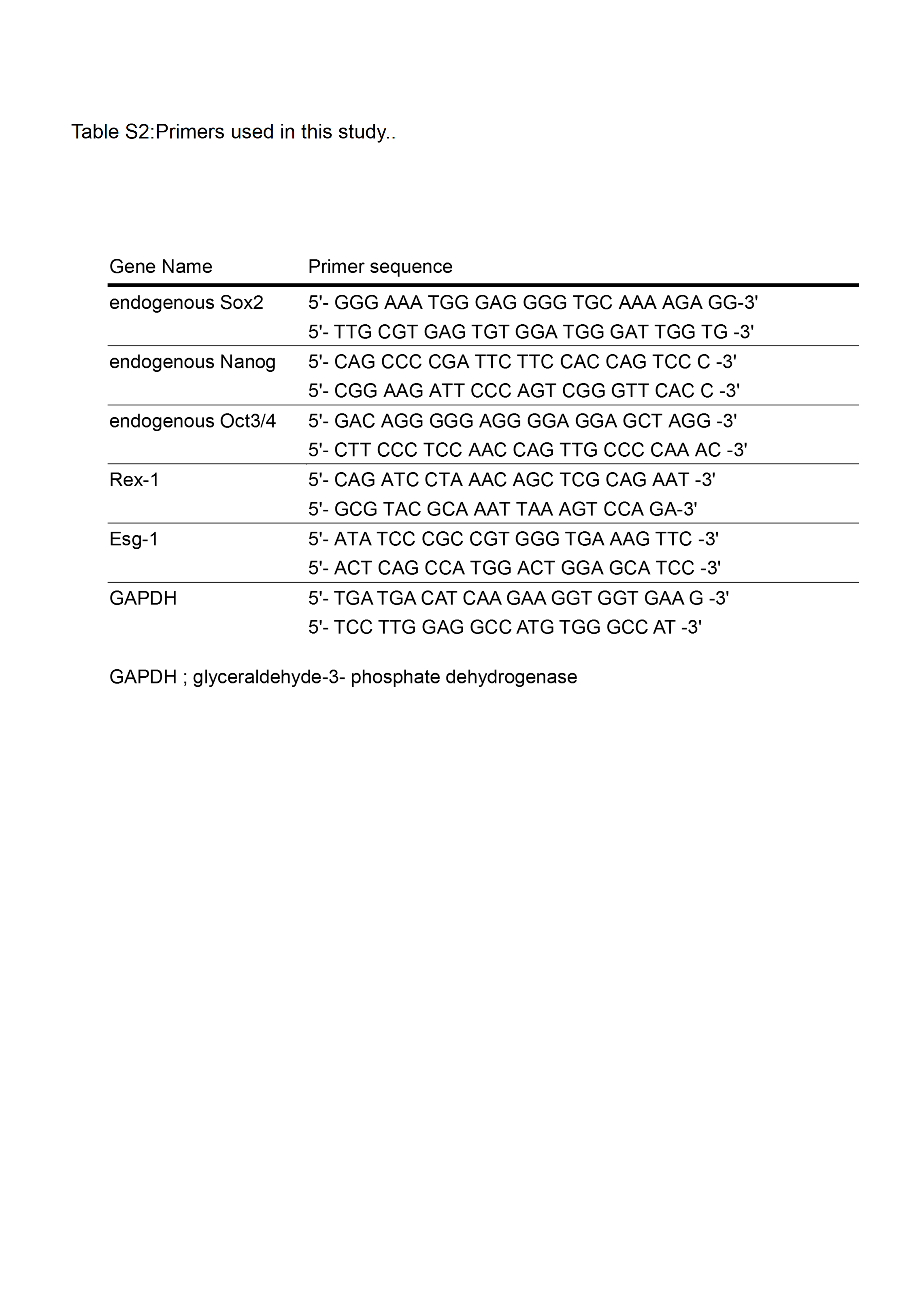

Supplement: Table S2 — Primers used in this study listed. (TIF) [file pone.0087151.s008.tif]

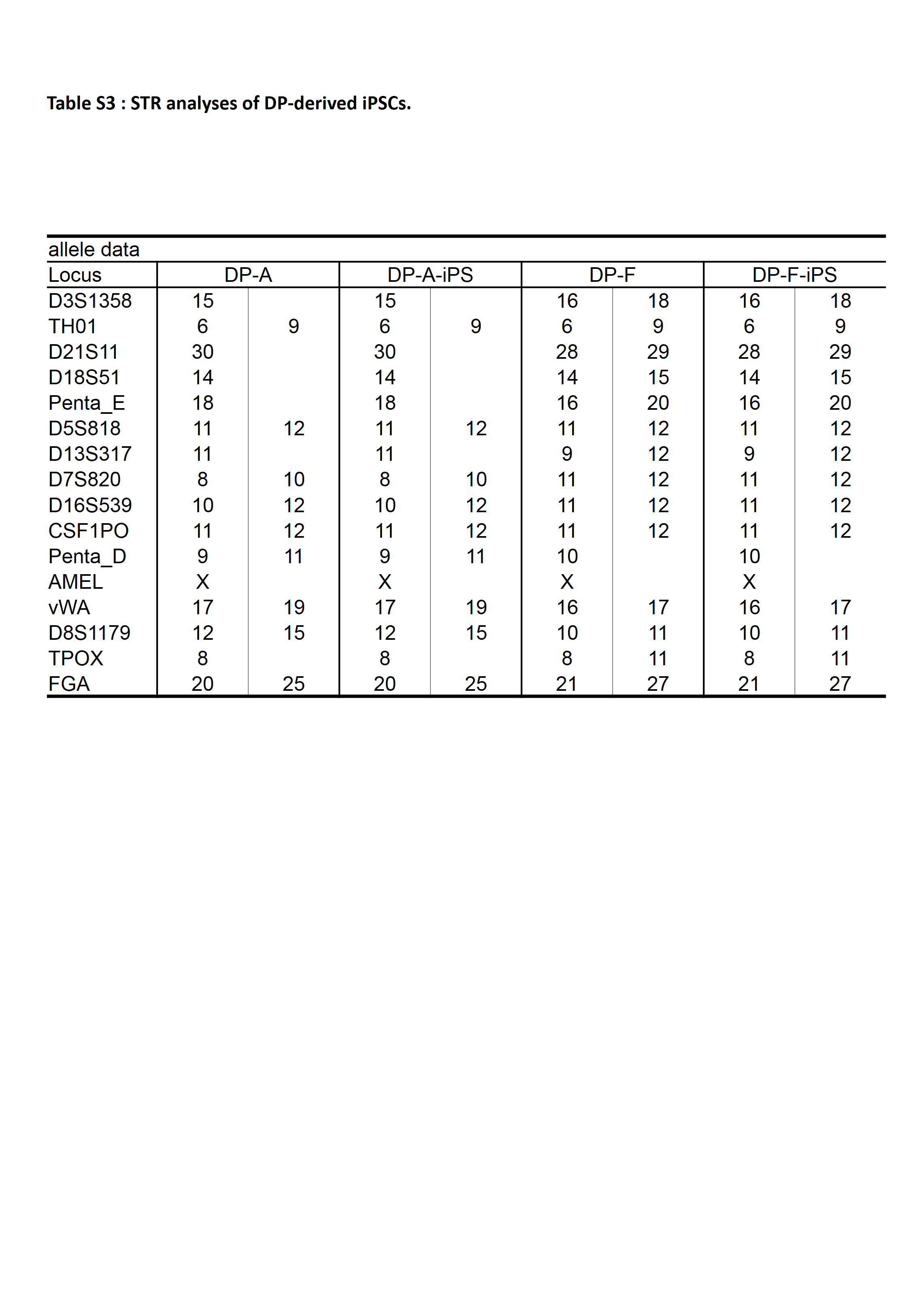

Supplement: Table S3 — STR analyses of DP-derived iPSCs. (TIF) [file pone.0087151.s009.tif]
