# Supplementary material for: Correction: Generation of Human Induced Pluripotent Stem (iPS) Cells in Serum- and Feeder-Free Defined Culture and TGF-β1 Regulation of Pluripotency
Source: PLoS One. 2014 Mar 18;9(3):e93242. doi: 10.1371/journal.pone.0093242 (PMC3958556; doi:10.1371/journal.pone.0093242)
Supplement: File S1 — Originally published, uncorrected article. [file pone.0093242.s001.pdf]

PLOS | ONE

Generation of Human Induced Pluripotent Stem (Ips) Cells in Serum- and Feeder-Free Defined Culture and TGF-Β1 Regulation of Pluripotency

Sachiko Yamasaki[1], Yuki Taguchi[1], Akira Shimamoto[2], Hanae Mukasa[1], Hidetoshi Tahara[2], Tetsuji Okamoto[1]*

1 Department of Molecular Oral Medicine and Maxillofacial Surgery, Applied Life Sciences, Graduate Institute of Biomedical & Health Sciences, Hiroshima University, Japan, **2** Department of Cellular and Molecular Biology, Basic Life Sciences, Graduate Institute of Biomedical & Health Sciences, Hiroshima University, Japan

Abstract

Human Embryonic Stem cells (hESCs) and human induced Pluripotent Stem cells (hiPSCs) are commonly maintained on inactivated mouse embryonic fibroblast as feeder cells in medium supplemented with FBS or proprietary replacements. Use of culture medium containing undefined or unknown components has limited the development of applications for pluripotent cells because of the relative lack of knowledge regarding cell responses to differentiating growth factors. In addition, there is no consensus as to the optimal formulation, or the nature of the cytokine requirements of the cells to promote their self-renewal and inhibit their differentiation. In this study, we successfully generated hiPSCs from human dental pulp cells (DPCs) using Yamanaka's factors (*Oct3/4, Sox2, Klf4*, and *c-Myc*) with retroviral vectors in serum- and feeder-free defined culture conditions. These hiPSCs retained the property of self-renewal as evaluated by the expression of self-renewal marker genes and proteins, morphology, cell growth rates, and pluripotency evaluated by differentiation into derivatives of all three primary germ layers *in vitro* and *in vivo*. In this study, we found that TGF-β1 increased the expression levels of pluripotency markers in a dose-dependent manner. However, increasing doses of TGF-β1 suppressed the growth rate of hiPSCs cultured under the defined conditions. Furthermore, over short time periods the hiPSCs cultured in hESF9 or hESF9T exhibited similar morphology, but hiPSCs maintained in hESF9 could not survive beyond 30 passages. This result clearly confirmed that hiPSCs cultured in hESF9 medium absolutely required TGF-β1 to maintain pluripotency. This simple serum-free adherent monoculture system will allow us to elucidate the cell responses to growth factors under defined conditions and can eliminate the risk might be brought by undefined pathogens.

Citation: Yamasaki S, Taguchi Y, Shimamoto A, Mukasa H, Tahara H, et al. (2014) Generation of Human Induced Pluripotent Stem (Ips) Cells in Serum- and Feeder-Free Defined Culture and TGF-Β1 Regulation of Pluripotency. PLoS ONE 9(1): e87151. doi:10.1371/journal.pone.0087151

Editor: Zhongjun Zhou, The University of Hong Kong, Hong Kong

Received August 30, 2013; **Accepted** December 19, 2013; **Published** January 29, 2014

Copyright: © 2014 Yamasaki et al. This is an open-access article distributed under the terms of the Creative Commons Attribution License, which permits unrestricted use, distribution, and reproduction in any medium, provided the original author and source are credited.

Funding: This work was supported in part by research grants (no. 22659369 and 24890139) from the Japanese Ministry of Education, Culture, Sports, Science and Technology to TO and SY. The funders had no role in study design, data collection and analysis, decision to publish, or preparation of the manuscript.

Competing Interests: The authors have declared that no competing interests exist.

* E-mail: tetsuok@hiroshima-u.ac.jp

Introduction

Human somatic cells can be reprogrammed into induced pluripotent stem cells (iPSCs) by introduction of transcription factors such as *Oct3/4*, *Sox2*, *Klf4* and *c-Myc* [1]. Embryonic stem cells (ESCs) and human iPSCs (hiPSCs) can proliferate without limit and yet maintain the potential to generate derivatives of all three germ layers. These properties make them useful for understanding the basic biology of the human body, for drug discovery and testing, and for transplantation therapies. However, the original protocol for the derivation of hiPSCs required feeder cells and mouse embryonic fibroblasts (MEF) to provide a microenvironment for the reprogramming and the maintenance of hiPSCs [1,2]. Although it is known that MEFs produce a number of secreted protein factors, they are traditionally used for ES cell culture. The inclusion of uncharacterized animal protein supplements makes culture conditions more complex with increased variability in nutrients and factors that contribute to cell growth and the maintenance of pluripotency. Furthermore there is unavoidable variability in using live cells as feeders, which may affect reprogramming steps. For these reasons defined culture conditions without feeder cells are desirable. Although several defined culture conditions without feeder cells for hiPSCs have been reported, manipulation of undifferentiated hESCs and hiPSCs still remains problematic. For example, as we show below, hiPSCs cultured in serum-free and feeder-free conditions in the absence of exogenous TGF-β1 lose pluripotency with passaging over time.

Previously, feeder-free methods using FGF-2 and activin A for iPS cell derivation from adult fibroblasts using hESF9 medium [3] (Table S1) or using chemically defined conditions for hiPS cell derivation and culture [4] have been reported. In the present study, we adapted a method that has been established for hES cell culture, which uses FGF-2 and heparin with a defined medium formulation [5]. We have validated the same formula in the reprogramming of fetal lung fibroblasts (TIG-3) and adult dental pulp cells (DPCs) to iPSCs without feeder cells. At first, we examined whether hESF9 medium and each of three ECM (type I collagen, gelatin, fibronectin) -coated surfaces could be used for iPS cell derivation. Although each ECM could generate hiPSCs,

type I collagen and gelatin could not maintain the pluripotency of hiPSCs. Secondly, we performed retrovirus production using PLAT-A cells in serum-free conditions and analyzed transduction efficiency. From these results, we performed hiPS cell generation from patient-derived DPCs using hESF9 medium with fibronectin in completely serum-free culture conditions. The medium is capable of maintaining reprogrammed cells that expressed ES cell factors and retained the potential to differentiate into all three embryonic germ layers.

TGF-βs and their family members have been implicated in the development and maintenance of various organs in which stem cells play important roles. In hESCs, the predominant signaling pathways involved in pluripotency and self-renewal are TGF-β, which signals through Smad2, 3, 4, and FGFR, which activates the MAPK and Akt pathways. Signaling through these pathways results in the expression and activation of three key transcription factors: Oct3/4, Sox2, and Nanog. These transcription factors activate gene expression of ESC-specific genes, regulate their own expression and also serve as hESCs markers. To improve the stability of hiPSC pluripotency, we investigated the effect of TGF-β1. The addition of TGF-β1 to the defined serum-free medium for hiPSCs supported the robust proliferation and continued pluripotency of hiPSCs. Here we show that hESF9 medium in completely defined serum-free culture conditions supports the derivation and maintenance of pluripotent stem cells. This culture system will allow us to elucidate the cell responses to growth factors under defined conditions. These advantages will be beneficial for clarifying the molecular mechanisms of early development.

Materials and Methods

Ethics Statement

Written approval for human tissue collection and subsequent iPS cell generation and genome/gene analyses performed in this study was obtained from the Ethics Committee for Human Genome/Gene Analysis Research at Hiroshima University (approval number: hi-58), and written informed consent was obtained from each individual patient. All animal experiments in this study strictly followed a protocol approved by the Institutional Animal Care and Use Committee of Hiroshima University (approval number: A-11-140).

Cell culture of Dental pulp cell

Using a protocol approved by the Ethics Committee for Human Genome/Gene Analysis Research at Hiroshima University, we collected normal human third molars at Hiroshima University Hospital after having obtained informed consent for the usage of dental pulp cells (DPCs) to derive iPSCs. Primary human dental pulp cell cultures were established from dental pulp tissue discarded during surgery. The pulp tissue samples were minced into small clumps and then transferred into type I collagen (0.15 mg/ml) (Nitta gelatin, Osaka, Japan)- coated culture dishes in RD6F serum-free medium [11,12]. The cells were cultured at 37°C in a humid atmosphere of 5% CO_2. Fibroblastic cells that grew out from these colonies were digested in 0.05% trypsin-ethylenediaminetetraacetic acid (EDTA) in Ca^{2+} and Mg^{2+}-free phosphate-buffered saline (PBS), and the trypsin was inactivated with 0.1% soybean trypsin inhibitor (Sigma Aldrich, St. Louis, MO) in PBS. These cells were subcultured every 2-3 day.

Retrovirus production using PLAT-A packaging cell line

PLAT-A packaging cells [6] (Cell Bio Labs Inc., San Diego, CA) were seeded at 2×10^6 cells on collagen-coated flasks and cultured overnight in DMEM supplemented with 10% FBS. The next day, pMXs retroviral vectors (Add Gene, Cambridge, MA) containing the open reading frames of *Oct3/4*, *Sox2*, *Klf4*,*c-Myc* and *EGFP* were transfected into PLAT-A cells with Xtreme GENE HP Transfection Reagent (Roche Diagnostics, Cambridge, MA). After 48 hr the medium was completely changed to serum-free hESF9. Viral supernatants were collected 48 h to 72 h after transfection, filtered through a 0.45 µm pore size PVDF filter (Millex-HV, Millipore, Billerica, MA) and supplemented with 8 µg/ml Polybrene (Sigma). The DPCs were transduced with *Oct3/4*:*Sox2*:*Klf4*:*c-Myc* (1:1:1:1) mixture of viral supernatant. To determine the viral transduction efficiency of individual factors, *EGFP* transduced retrovirus supernatant was transduced to DPCs. Medium was changed every other day, and the cells cultured for 4 days. The cells were trypsinized and analyzed by flow cytometry (FACS Calibur™) (BD Biosciences, San Jose, CA).

The generation of hiPS cell using TIG-3 under feeder- and serum-free, defined culture conditions from the reprogramming step

To obtain iPSCs, TIG-3 (derived from fetal lung fibroblasts and purchased from the Health Science Research Resources Bank, Osaka, Japan) [7] cultured in DMEM supplemented with 10% FBS were transduced with the pMXs-based retroviral vectors encoding human *Oct3/4*, *Sox2*, *Klf4* and *c-Myc*, as described above. At the same time, TIG-3 were transduced the EGFP-expressing retroviral vector or the control vector with a constant amount of total DNA. After 4 days cells were photographed under a fluorescence microscope and analyzed by flow cytometry (FACS Calibur™). After 4 days cells transduced with the four factors were trypsinized and plated on 0.1% gelatin- (Millipore), or type I collagen- (0.3 mg/ml) (Nitta gelatin) or fibronectin- (2 µg/cm^2) (Sigma) coated dishes in hESF9 medium. For comparison, we used KSR-based medium and mitomycin C-treated MEF (Embryo Max® PMEF-H, Millipore) [8–10] as feeder cells (KSR-based conditions)[1,2]. After 20 days, we detected colonies that were subsequently passaged and maintained in hESF9 medium with individual ECMs. After 36 days of culture, ALP–positive colonies were counted.

Retrovirus Production using PLAT-A cell in serum-free conditions and transduction efficiency

Retroviral supernatants of pMXs-(empty) and pMXs-(EGFP) were produced in PLAT-A packaging cells in hESF9 medium or DMEM supplemented with 10% FBS. These collected virus supernatants were used for infection. After 3 days, infected TIG-3 cells were photographed under a fluorescence microscope and transduction efficiency analyzed by FACS Calibur™ of EGFP expression.

Generation of hiPS Cells from DPCs in completely defined culture conditions

DPCs were seeded at 3×10^5 cells per 60-mm dish in RD6F serum-free medium [11,12] and cultured overnight. The next day the cells were infected with viral supernatant for 24 h in hESF9 medium. Four days after transduction, these infected cells were harvested by trypsinization and seeded on fibronectin (2 µg/cm^2) (Sigma F-1141) -coated dishes at 1×10^5 cells per 100 mm dish in hESF9-medium. The medium was changed every other day. Approximately 20 days after infection, iPS colonies were picked based on human ES cell-like colony morphology. The picked colonies were subsequently expanded and maintained on fibronectin in hESF9T medium. Reprogramming efficiency was determined as the positive number of total ES-like ALP positive

colonies per total number of infected cells. Thirty-three days after transduction, we detected hiPSC-like colonies by ALP substrate staining. As a control, transduced DPCs were seeded on mitomycin-C-treated MEF feeder cells with KSR-based conditions [1,2].

Maintenance of human iPS cells in serum-free culture conditions

For subculturing colonies were mechanically detached from the culture dish and dissociated into small clamps by pipetting. The cell suspension was transferred on fibronectin-coated dishes in hESF9 medium or hESF9 with TGF-β1 (2 ng/ml) (R&D systems, Minneapolis, MN) (hESF9T). We defined this stage as passage 1. The medium was changed daily with hESF9T medium.

Cell growth analysis of human iPS cells generated and maintained in define culture conditions

Human iPSCs generated under hESF9 and cultured in hESF9T (DP-F-iPS-CL8 passage 38, DP-F-iPS-CL4 passage 38, DP-F-iPS-CL16 passage 33) were seeded in a 24-well plate coated with fibronectin and counted every 24 hr. Growth curves were calculated from each passage split ratio. The hiPSC colonies (DP-F-iPSCs) cultured for 1 month in feeder-free hESF9 or hESF9T were detached using a cell scraper and 0.001% trypsin-0.01% ethylenediaminetetraacetic acid (EDTA). The dissociated cells were then fixed, incubated with Alexa Fluor 647®-conjugated SSEA4 antibody or PerCP-Cy5.5-conjugated Oct3/4, and subjected to flow cytometry (FACS Aria™).

Alkaline phosphate (ALP) staining and Immunocytochemistry

Alkaline phosphatase staining was performed using a Fast Red substrate kit (Nichirei Biosciences Inc., Tokyo, Japan). To detect pluripotent stem cell marker antigens cells were fixed with PBS containing 4% paraformaldehyde for 10 min at room temperature. After washing with PBS, the cells were treated with PBS containing 5% normal goat serum (Nichirei) and 0.1% Triton X-100 for 45 min at room temperature. Fixed cells were stained with primary antibodies included SSEA-4 (1:100, Stemgent®, Cambridge, MA), TRA-1-60 (1/200, Stemgent®), TRA-1-81 (1/200, Stemgent®), Oct-3/4 (1/200 Millipore), Nanog (1/600, ReproCELL, Yokohama, Japan), Nestin (1/200, Millipore), βIII-tubulin (1/200, Millipore), α-smooth muscle actin (pre-diluted, DAKO Cytomation, Glostrup, Denmark) and α-fetoprotein (1/100, R&D Systems). These primary antibodies were visualized with Alexa Fluor® 488- conjugated goat anti-rabbit IgG, or Alexa Fluor® 594-conjugated goat anti-rabbit IgG, or Alexa Fluor® 488-conjugated goat anti-mouse IgG, or Alexa Fluor® 594-conjugated goat anti-mouse IgG (1/200, Invitrogen, Carlsbad, CA). Nucleuses were stained with DAPI. Fluorescence images were acquired using a Zeiss inverted LSM confocal microscope (Carl Zeiss, GmbH, Germany).

RNA isolation and reverse transcription gene expression

A detailed reverse transcription-polymerase chain reaction (RT-PCR) protocol was described previously [13]. Briefly, total RNA was extracted from iPSCs using the Illustra RNA spin Mini Isolation kit (GE Healthcare UK Ltd, Buckinghamshire, England), according to manufacturer's instructions. cDNA was synthesized from 1 μg of total RNA using High capacity RNA-to cDNA master mix (Applied Biosystems, Carlsbad, CA). RT-PCR was performed with AmpliTaq Gold DNA polymerase with Gene Amp (Applied Biosystems). The primers used in this study are described in Table S2. PCR products were size-fractionated using 1.5% agarose gel electrophoresis. DNA markers were used to confirm the size of the fragments.

Droplet digital PCR analysis

Droplet digital PCR (ddPCR) analysis was performed using QX100™ Droplet Digital™ PCR (Bio-RAD Laboratories, Hercules, CA). Total RNA was extracted from hiPSCs, and RT-PCR was performed. cDNA samples, primers and probes with the ddPCR master mix (Bio-Rad) were combined in water-oil emulsion droplets. These droplets were subjected to 40 PCR cycles. Positive and negative fluorescent droplets in each sample were detected with a QX100 Droplet reader (Bio-Rad). The relative mRNA expression in each sample was normalized to its GAPDH content. The mRNA levels in each cells were expressed relative to those in hESF9 medium (TGF-β1; 0 ng/ml), which was taken as 1. The results are presented as means±SD of three independent experiments.

Embryoid body formation

In vitro differentiation was induced by the formation of embryoid bodies as described previously [5]. Briefly, undifferentiated human DP-iPSCs were cultured in DMEM with 10% FBS for 4 days in low-attachment 96 well plates. After 4 days in suspension culture, floating embryoid bodies were re-seeded onto gelatin-coated dishes in the same culture medium for 10 days. The medium was changed every other day.

Teratoma formation assay and histological analysis

Human DP-iPSCs were suspended at 2×10^7 cells/ml in PBS and injected 50 ul of the cell suspension (1×10^6 cells) subcutaneously into dorsal flank of SCID (CB17/Icr-*Prkdc*^{scid}/CrlCrlj) mice. Ten weeks after the injection, tumors were surgically dissected from the mice. Teratomas were weighed, fixed in PBS containing 4% formaldehyde, and embedded in paraffin. Sections were stained with hematoxylin and eosin and Alcian Blue stain.

Short tandem repeat DNA analysis

Genomic DNA was used for PCR with Powerplex 16 system (Promega Corporation, Madison, WI) and analyzed by ABI PRISM 3100 Genetic analyzer and Gene Mapper v3.5 (Applied Biosystems).

RNA expression array analysis

A heat map generated from the RNA expression array data displayed the expression profile of the hESC-enriched genes and the differentiated cell-enriched genes. The genes shown in blue represented the down-regulation of gene expression, whereas the genes shown in red represented the up-regulation of gene expression. RNA expression array were performed using the Agilent Sure Print G3 Human GE 8x60K v2 Microarray, and data were analyzed using Genespring12.0 (Agilent Technologies, Santa Clara, CA).

Results

The generation of hiPS cells using TIG-3 under feeder- and serum-free, defined culture conditions from the reprogramming step

We examined whether hESF9 medium and each of three ECM (type I collagen, gelatin, fibronectin)-coated surfaces could be used for iPS cell derivation. We used Platinum-A retroviral packaging cell, amphotropic (PLAT-A) [6] carrying the *Oct3/4, Sox2, Klf4*

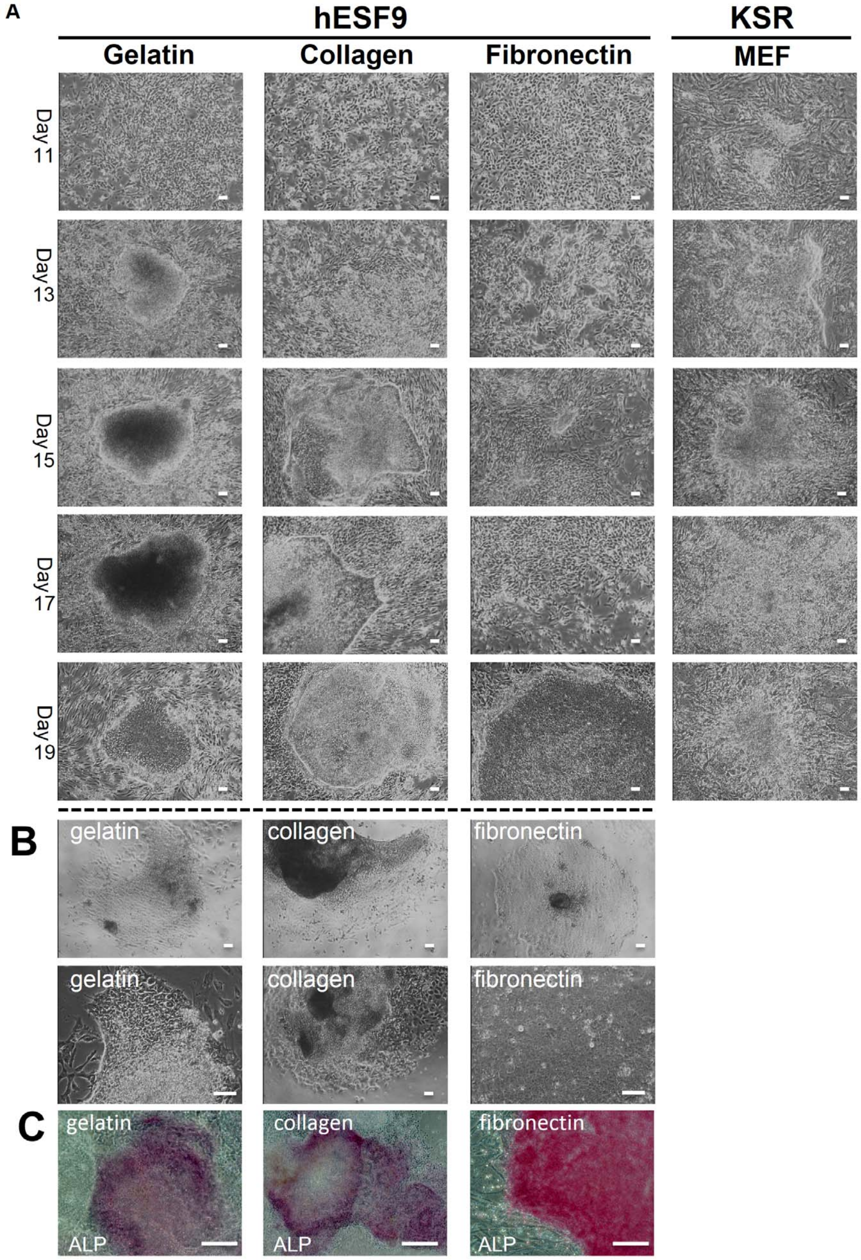

Figure 1. Morphology of transduced TIG-3 on each ECMs in hESF9 medium. A) Transduced TIG-3 cells were cultured on each ECMs with hESF9 medium or on MEF with KSR-based conditions. After 20 days, iPS colony were picked up and sub-cultured on each ECMs. B) Images of sub-cultured iPS colonies seeded after 2days on each ECMs with hESF9 medium. C) ALP staining of iPSCs on gelatin, collagen, and fibronectin (infect after 36days). Bars indicate 200 μm.
doi:10.1371/journal.pone.0087151.g001

and *c-Myc* in DMEM supplemented with 10% FBS. We produced retroviruses using PLAT-A cell line in serum-supplemented conditions as described in the manufacture's protocol. Then we transduced four factors (*Oct3/4, Sox2, Klf4* and *c-Myc*) into TIG-3 (normal fibroblast cell line derived from fetal lung) [7] in DMEM supplemented with 10% FBS. At the same time, we transfected the EGFP-expressing retroviral vector and the empty control vector into TIG-3 with a constant amount of total DNA. After 4 days, cells were photographed under a fluorescence microscope and analyzed by flow cytometry. The transduction efficiency of the four factors was 26.6% as measured by EGFP expression (Fig. S1). After 4 days four factor-transduced cells were trypsinized and plated on 0.1% gelatin, or type I collagen (0.3 mg/ml) or fibronectin (2 $\mu g/cm^2$) in hESF9 medium. For comparison, we used KSR-based medium and mitomycin C-treated MEF [8-10] feeder cells (KSR-based conditions) [1,2]. After 13 days of culture, we observed human ES-like colonies, characterized by large nuclei and little cytoplasm (Fig. 1-A). After 20 days colonies were passaged and maintained in hESF9 medium on substrates coated with individual ECMs (Fig. S2-A). After 36 days of culture, alkaline phosphatase (ALP)–positive colonies were counted, and relatively high iPS induction efficiency was observed. However, although type I collagen supported hiPS cell generation more effectively than the other two ECMs, collagen and gelatin did not

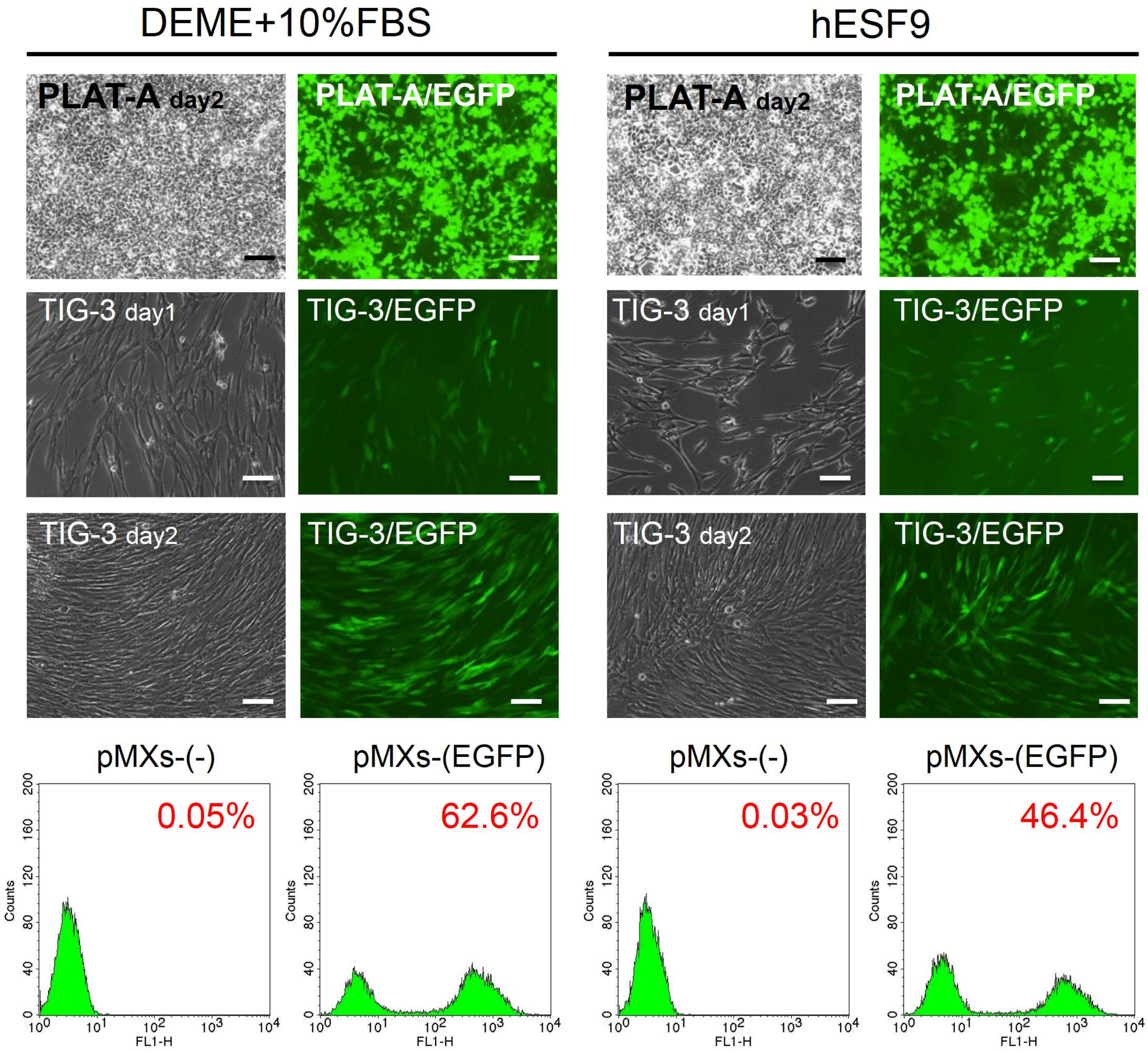

Figure 2. Transduction efficiency of retroviruses in serum-free hESF9 medium and serum-supplemented medium in TIG-3. TIG-3 was introduced with pMXs retroviruses containing the EGFP cDNA. After 3 days, cells were photographed under a fluorescence microscope and analyzed by flow cytometry. The left panel shows the images of phase contrast, fluorescent microscope and the results of flow cytometry of the cells cultured in serum-supplemented condition (DMEM+10%FBS). The right panel shows the cells in serum-free culture conditions (hESF9). Transfection efficiency of EGFP was 62.6% in serum-supplemented condition and 46.4% in serum-free culture condition. Bars indicate 200 μm.
doi:10.1371/journal.pone.0087151.g002

support pluripotency of hiPSCs after further culture of these colonies (Fig.1-B). This inability to maintain pluripotency of hiPSCs was confirmed by ALP activity (Fig.1-C) and RT-PCR analysis (Fig. S2-B).

Retrovirus production using PLAT-A cell in serum-free conditions and analysis of transduction efficiency

We examined whether the serum-free culture condition could produce relatively high titer viral supernatants. Retroviral supernatants of pMXs-(empty) and pMXs-(EGFP) were produced in PLAT-A packaging cells in serum-free hESF9 medium or serum supplemented medium. The collected virus supernatants were used for infection. After 3 days, transduction efficiency was measured by FACS analysis of EGFP expression. Transduction efficiency in serum-free hESF9 medium was 46.4%, whereas it was 62.6% in serum-supplemented medium (Fig. 2, Fig. S3). While the data showed a slightly lower titer in hESF9, the viral titer was sufficient to reprogram dental pulp cells (see below).

hiPS cell generation from adult DPCs in serum- and feeder-free culture condition

We obtained dental pulp cells (DPCs) from healthy patients (Fig. 3, Fig. S4). These DPCs were reprogrammed to hiPSCs after transduction with PLAT-A virus in completely serum-free culture conditions (Fig. 3). The transduced DPCs were trypsinized and plated on fibronectin-coated dish in hESF9 medium. Human iPS

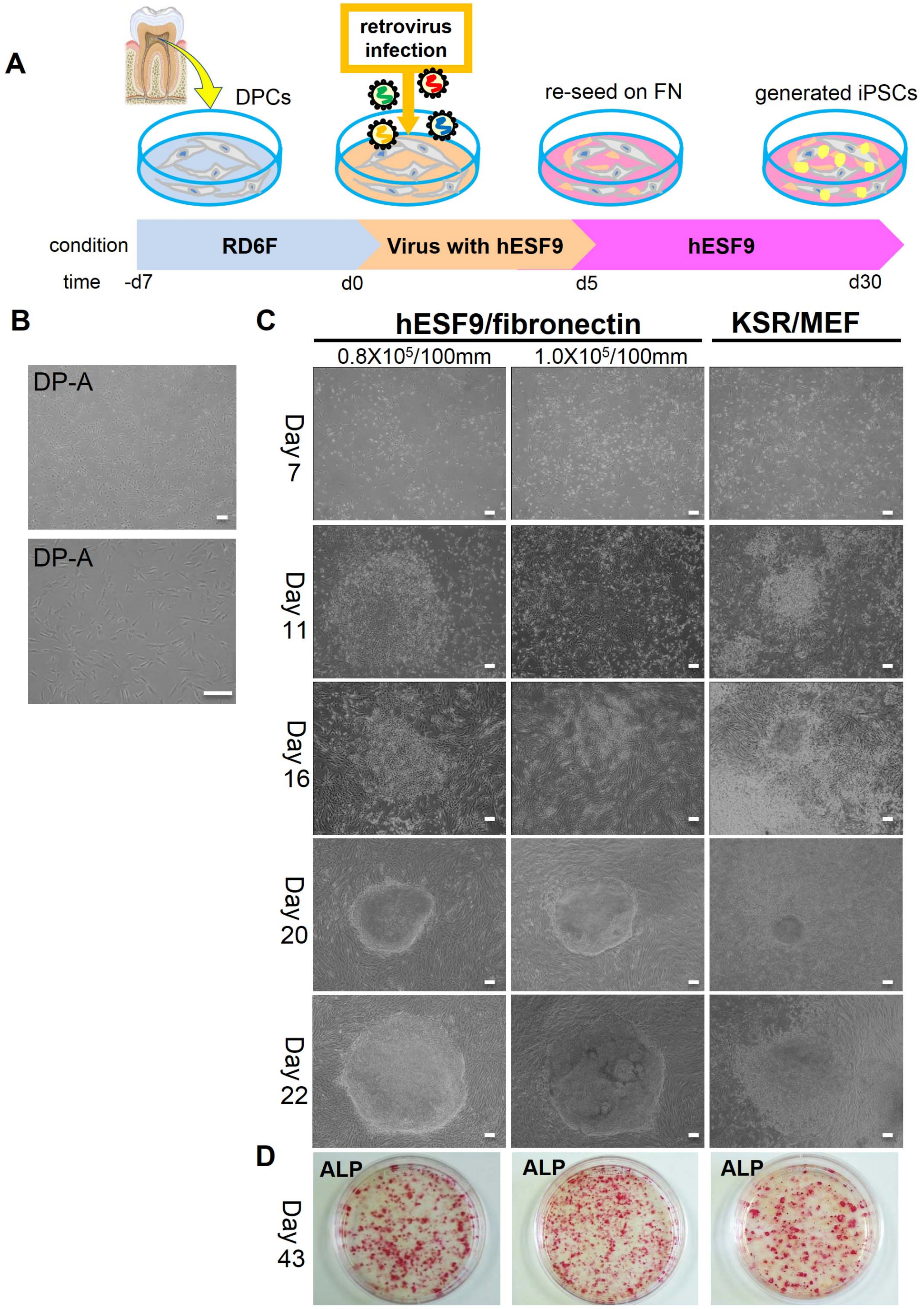

Figure 3. hiPSC generation from DPCs in serum- and feeder-free culture conditions. A) Time schedule of hiPSC generation. Day-7~0: DPCs were cultured in RD6F serum-free medium on type I collagen-coated dish. Day 0~4: Retroviral transduction (*Oct3/4, Sox2, KLF-4, c-Myc*) with hESF9 medium. Day5: re-seeding on fibronectin-coated plate with hESF9 medium. Day6~30: replace medium every other day. B) Images of DPCs (DP-A: passage 4) on type I collagen-coated plate with RD6F medium. C) Transduced DPCs were cultured on fibronectin with hESF9 medium or on MEF with KSR-based conditions. After 20 days, iPS colony were picked up and sub-cultured on fibronectin. The reprogramming efficiency was 0.23–0.38% with a high success rate. D) ALP staining of iPSCs on fibronectin, 43 days after infection. Bars indicate 200 µm.
doi:10.1371/journal.pone.0087151.g003

cell colonies intermingled with partially reprogrammed colonies appeared at 15 days after transfection. After 20 days, individual iPS cell colonies were selected and subsequently passaged and maintained in hESF9 medium with TGF-β1 (2 ng/ml) (hESF9T) in dishes coated with fibronectin (Fig. 3-C). After 33 days in culture, ALP-positive colonies were counted; the efficiency of reprogramming was 0.23–0.39%. By contrast, iPS cell colonies did not emerge at all when cells were not re-plated on fibronectin after viral infection (data not shown). When TGF-β1 was present throughout the reprogramming procedure using hESF9T medium, the number of iPS colonies were 10-fold lower than that in

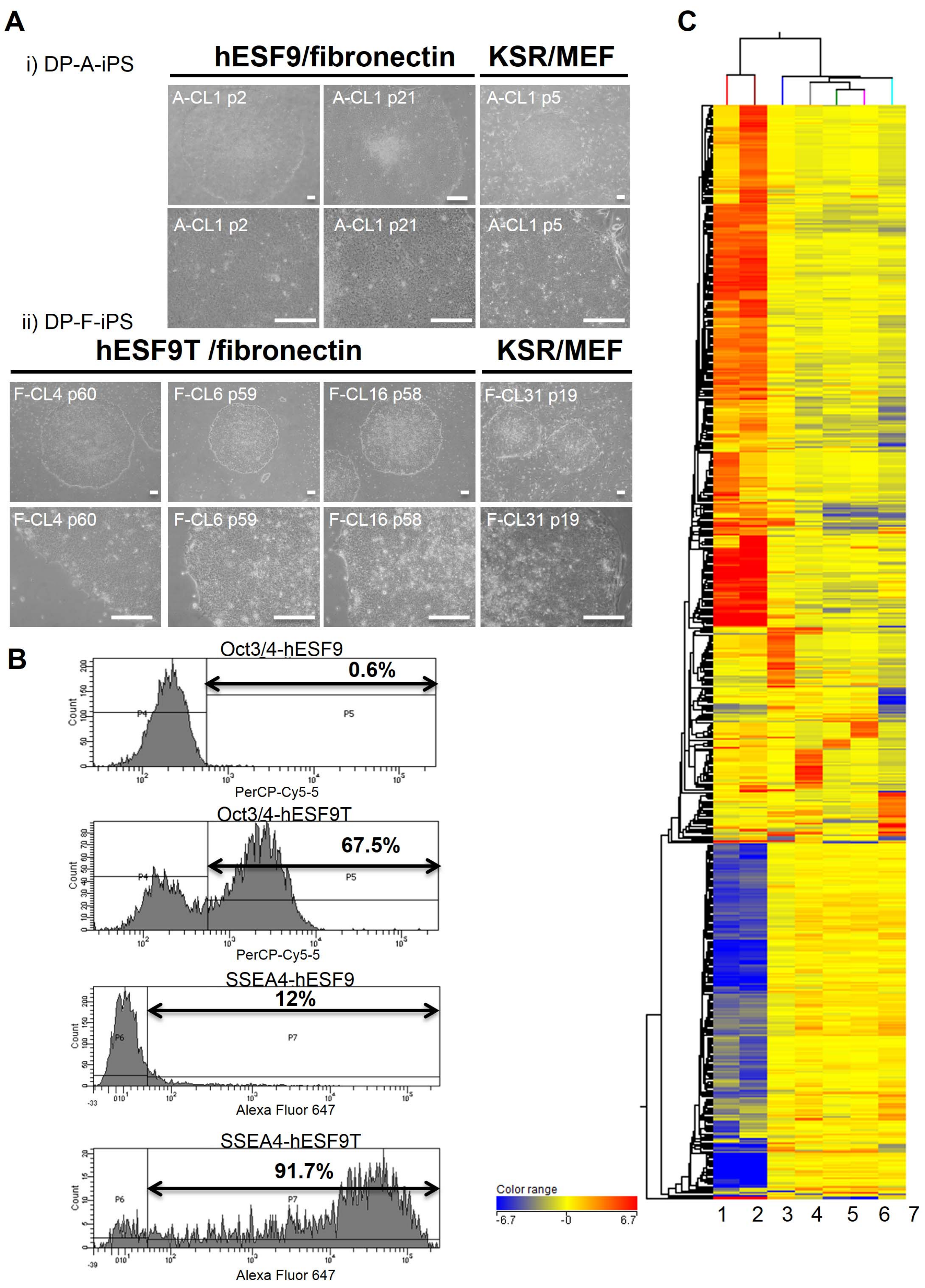

Figure 4. hiPSCs derived from DPCs in completely serum- and feeder-free culture conditions. A) Phase contrast images of iPSCs derived from DPCs (DP-A-iPS and DP-F-iPS). i) DP-A-iPS-CL1 at passage 2, or 21 on fibronectin-coated dish with hESF9 medium. Right panel showed the cells at passage 5 cultured on MEF with KSR-based conditions. ii) DP-F-iPS-CL4 at passage 60, CL6 at passage 59 and CL16 at passage 58 on fibronectin-coated dish with hESF9T medium. Right panel showed CL31 at passage 19 on MEF with KSR-based conditions. Bars indicate 200 μm. B) Flow cytometry analysis of Oct3/4 and SSEA-4 expression in hiPSCs generated and maintained in hESF9 medium supplemented with TGF-β1 (2 ng/ml) (hESF9T) or without TGF-β1 (hESF9) (DP-F-iPS-CL-8 at passage 33). The horizontal bar indicates the gating used to score the percentage of cells antigen positive. C) Comparison of the global gene expression analysis. Unsupervised clustering was performed using microarray data from parental cell (DPCs), DP-iPSCs cultured in hESF9 or hESF9T (DP-A-iPS, DP-F-iPS) and hiPSCs (Tic, DP-F-iPS). 1.DP cell (DP-A): passage 2 = before infection. 2.DP

cell (DP-F): passage 4 = before infection. 3.DP-A-iPS-CL1: passage 14 = serum-free condition (hESF9/on FN). 4.DP-F-iPS-CL12: passage 36 = KSR-based condition (KSR/on MEF). 5.DP-F-iPS-CL6: passage 37 = serum-free condition (hESF9T/on FN). 6.DP-F-iPS-CL8: passage 35 = serum-free condition (hESF9T/on FN). 7.Tic (hiPSC: JCRB1331): passage 58 = KSR-based condition (KSR/on MEF). A genome-wide gene expression profiling analysis demonstrated that hiPSCs cultured in hESF9 or hESF9T on fibronectin showed a similar gene expression pattern to those grown in a conventional feeder-dependent culture (KSR-based condition). Hierarchical combined tree on compare. (Fold change> = 20).
doi:10.1371/journal.pone.0087151.g004

hESF9 medium and DPCs tended to overgrow and inhibit the emergence of iPS colonies (data not shown).

TGF-β1 plays an important role in maintaining the pluripotency of hiPSCs

Newly generated hiPSCs were mechanically detached from culture dishes and transferred to fibronectin-coated dishes in hESF9 or hESF9T (containing TGF-β1) medium (Fig. 4-A). In hESF9 medium, hiPSCs attached to the dish, but they did not remain undifferentiated, and we occasionally observed spontaneous differentiation along the edges of colonies. Small differentiated cells were observed in hESF9 medium in the absence of TGF-β1. By contrast, hiPS colonies remained undifferentiated in hESF9T medium.

Increasing the dose of TGF-β1 up to 10 ng/ml promoted the growth of undifferentiated colonies as confirmed by QX100™ Droplet Digital™ PCR (Bio-RAD) analysis (Fig. 5, A-C). The greatest effect of TGF-β1 was seen at 2–10 ng/ml, whereas 0 ng/ml was markedly deleterious. The expression of pluripotency markers such as Nanog and Oct3/4 were up-regulated by TGF-β1. Moreover, the expression of differentiated marker genes was lower in hESF9T than in hESF9 medium (Fig.5-B). Plasminogen activator inhibitor-1 (PAI-1) is an indicator of mesoderm differentiation, and GATA binding protein 4 (GATA4) is a marker involved in the development of cardiac hypertrophy and remodeling, and it plays a critical role in regulating basal and agonist or stress-induced gene expression in cardiac and smooth muscle cell types. At passage 33, the percentage of SSEA4-positive-DP-F-iPS-CL8 cells cultured in hESF9T (91.7%) was higher than that in hESF9 medium (12%). Moreover, the percentage of Oct3/4-positive-DP-F-iPS-CL4 cells was 67.5% in hESF9T medium and 0.6% in hESF9 medium (Fig. 4-B). These results indicated that TGF-β1 supported to a large extent the undifferentiated growth of hiPSCs over a prolonged period. The hiPSCs generated and maintained in hESF9 did not survive beyond 30 passages. We continued to culture human iPSCs in hESF9T up to 60 passages.

Gene expression analysis confirmed the effect of TGF-β1 in maintaining pluripotency of hiPSCs

A genome-wide gene expression profiling analysis demonstrated that hiPSCs cultured in hESF9 or hESF9T on fibronectin showed broadly similar gene expression patterns to those grown in a conventional feeder-dependent culture (KSR-based condition) (Fig. 4-C, Fig. S5). In contrast, the cells cultured in hESF9 exhibited significantly different profiles in several signaling pathways from those cultured in hESF9T. In a pathway analysis the TGF-β signaling pathway (WP560), the WNT signaling and pluripotency pathway (WP399), the WNT signaling pathway (WP428), and Apoptosis modulation and signaling (WP1772) displayed significant differences between hESF9T and hESF9 (data not shown). Thus, DP-iPSCs cultured in hESF9T for a prolonged period remained undifferentiated and exhibited a similar gene expression pattern as cells grown in conventional feeder dependent cultures.

Characterization of DPC- derived iPS cells

An hiPS clone cultured in hESF9 or hESF9T showed a characteristic human ES cell-like morphology (Fig. 4-A), and reactivation of endogenous pluripotency marker genes such as *Oct3/4, Sox2, Nanog, Esg1,* and *Rex-1* was detected by RT-PCR (Fig. 5-D). These cells exhibited ALP activity and expressed SSEA-4, Tra-1-60, Tra-1-81, Nanog and Oct3/4 (Fig. 5-E). We confirmed the differentiation potential of the cells using an *in vitro* differentiation assay involving embryoid body generation. After 14 days of differentiation culture, the embryoid bodies contained a variety of differentiated cells characterized by germ-layer markers. These induced populations of cells were immunoreactive with antibodies to Nestin and βIII-tubulin (ectoderm markers), α-smooth muscle actin (SMA) (mesoderm marker), and α-fetoprotein (AFP) (primitive endoderm marker), but they did not react with anti-Oct3/4 (Fig. 6-A). The pluripotency of the iPS cell clone was also confirmed by the presence of cell derivatives of all three germ layers by teratoma formation after injection of undifferentiated iPS cells into severe combined immunodeficient (SCID) mice. Ten weeks after injection, histological analysis demonstrated that the formed tumors were derived from all three germ layers (n = 3). Neural tissues (ectoderm), epithelium (ectoderm), muscle (mesoderm), cartilage (mesoderm), adipose (mesoderm) and intestinal epithelial tissues (endoderm) were identified histologically in the hiPSCs-derived teratomas (Fig. 6-B).

Short Tandem Repeat Analysis

The genetic identity of DPCs and generated iPSCs was proven by a short tandem repeat analysis of genomic DNA (Table S3).

Cell growth and karyotype analysis of human iPS cells generated and maintained in define culture conditions

Growth curves were calculated from the split ratios at each passage. The population doubling time was 16.6±0.8 h (Fig. S6-A). The generated hiPSCs also had the property of self-renewal and pluripotency, and they possessed a normal karyotype. Karyotype analysis revealed that iPSCs at passage 20 were 46, XX (Fig. S6-B).

Discussion

We have established a fully defined serum-free culture system for the purposes of standardizing culture methods and protocols for deriving hiPSCs. Previously, we have demonstrated a defined serum- and feeder-free culture system based on use of hESF9 medium without TGF-β1 for human ES cell culture [2,5]. The hESF9 medium consists of a basal nutrient medium with known protein components, and it thus reduces the risk of contamination from adventitious pathogens. In this study, we showed that hiPSCs can be generated and maintained in a fully defined serum-free culture system from primary cell cultures of patient samples. The established hiPSCs are similar to hESCs in many respects, including morphology, proliferation, surface markers, gene expression, in vitro differentiation, and teratoma formation.

We first identified serum-free culture conditions that supported iPS cell generation. Several animal product-free culture media have been reported to support the derivation and/or maintenance

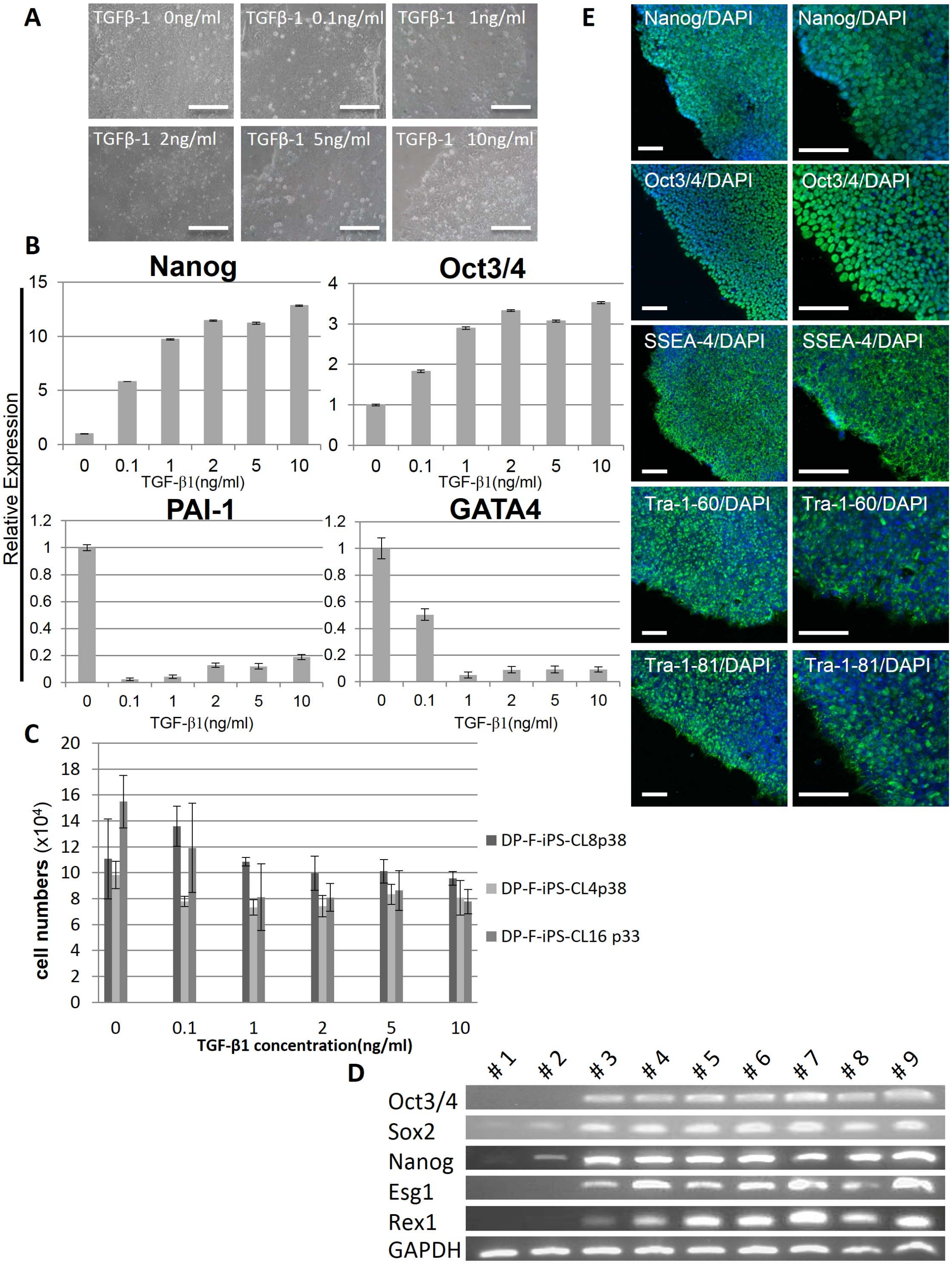

Figure 5. Culture of hiPSCs in hESF9T and Self-renewal marker expression of pluripotent stem cells in hiPSCs in defined culture conditions. A) Phase contrast photomicrograph of DP-F-iPS-CL16 (passage 28) supplemented with various concentration of TGF-β1 (0, 0.1, 1, 2, 5, 10 ng/ml). B) Digital-PCR analysis of gene expression of Nanog, Oct3/4, PAI-I and GATA4 in DP-F-iPS-CL6 in hESF9 medium supplemented with TGF-β1 (0, 0.1, 1, 2, 5, 10 ng/ml) on fibronectin. Expression levels were all normalized against GAPDH. C) Effects of TGF-β1 on hiPS cell proliferation. hiPSCs generated under hESF9 and cultured in hESF9T (CL-4 passage 38, CL-8 passage 38, CL-16 passage 33) were seeded in a 24 well plate coated with fibronectin at 1×10^4 cells/well and counted at every 24 hr. Each bar shows the number of cells in each concentration of TGF-β1 after 6 days of culture. Increasing the dose of TGF-β1 up to 10 ng/ml suppressed the growth of hiPSCs. Bars represent the mean±SEM. (n =3). D) Expression of ES cell marker genes in iPSCs derived from DPCs. We used primers that only amplified the endogenous genes. #1: DP cell (DP-A): passage 2 = before infection. #2: DP cell (DP-F): passage 4 = before infection. #3: DP-A-iPS-CL1: passage 14 = serum-free condition (hESF9/on FN). #4: DP-F-iPS-CL4: passage 37 = serum-free condition (hESF9T/on FN). #5: DP-F-iPS-CL6: passage 35 = serum-free condition (hESF9T/on FN). #6: DP-F-iPS-CL8: passage 35 = serum-free condition (hESF9T/on FN). #7: DP-A-iPS-CL1: passage 8 = KSR-based condition (KSR/on MEF). #8: DP-F-iPS-CL12: passage 36 =

KSR-based condition (KSR/on MEF). #9: Tic (hiPSC: JCRB1331): passage 103 = KSR-based condition (KSR/on MEF). E) Immunocytochemistry of Nanog, Oct3/4, SSEA-4, Tra-1-60 and Tra-1-81. DP-F-iPS-CL16 grown under hESF9T-based culture conditions for 19 passages were fixed and reacted with antibodies (Nanog, Oct3/4, SSEA-4, Tra-1-61 and Tra-1-81). Binding of these antibodies was visualized with Alexa Fluor® 488-conjugated secondary antibodies (green). Nuclei were stained with DAPI (blue). Scale bars represent 100 μm.
doi:10.1371/journal.pone.0087151.g005

of hESCs, but their performance tends to be lower than that of KSR-based medium [14]. Our data showed that iPSCs can be generated in serum-free hESF9 medium by retroviral transduction of four transcription factors, *Oct3/4*, *Sox2*, *Klf4*, and *c-Myc* cultured on type I collagen or gelatin or fibronectin, and the absence of serum did not affect the efficiency of cell reprogramming. Subsequently, we demonstrated that serum-free medium also did not reduce retroviral transduction efficiency. In this study, we demonstrated that individual attachment factors could support generation of hiPSCs in place of MEF feeder cells. We showed that gelatin, collagen and fibronectin enabled rapid and steady generation of hiPSCs. However, once generated, iPS cells subcultured on gelatin or collagen did not retain their pluripotency, and these cells began to undergo differentiation.

It has been reported that fibronectin supports the maintenance of hESCs via α5β1 integrin [15], and Ras/MEK/MAPK signaling and kinases were stimulated via integrin ligation. Several groups have reported that vitronectin and one of its variants (VTN-NC) supports the maintenance of hESCs via αVβ5 integrin [4,16,17]. Another group has reported that the adhesion of hESCs and hiPSCs to laminin-511 is maintained via integrin α6β1, and Akt/ERK and kinases interacting with FAK are highly phosphorylated in human pluripotent stem cells [18–20]. These studies show that stem cell–ECM interactions are important in maintaining stem cell adhesion, survival and self-renewal both *in vivo* and *in vitro*.

In this study, we showed that individual ECM components differentially affected cell morphology and the efficiency of iPS induction through signals transmitted to the cell from the extracellular environment. The maintenance and proliferation of hiPSCs require a "niche" micro-environment [21]. Pluripotency was affected by many growth factors and cytokines interacting with cells. In the course of this process the acceleration of cell proliferation caused by cell cycle regulation is also important. The proliferation rate of ESCs is very rapid. The transduced cells acquired ESC-like characteristics, consequently the number of reprogrammed "iPSCs" increased. Moreover, the majority of transduced cells were incompletely reprogrammed and consequently did not generate ES cell-like colonies. Only a small proportion of transduced cells were able to form human ES cell-like colonies. It is unknown whether incompletely reprogrammed fibroblast-like cells supported the formation of ES cell-like colonies that emerged within fibroblast-like colonies through a feeder-like effect or through a non-autonomous effect. We believe that further studies using a fully defined medium will lead to a clarification of the reprogramming mechanisms and the advancement of stem cell research. This medium will greatly reduce variations in culture conditions arising from undefined medium constituents.

It is believed that the signaling requirements of human iPS cell generation and maintenance are different. In this report we have shown that hESF9 medium containing FGF-2 and heparin enhanced the derivation of iPSCs. However, the same conditions did not maintain the pluripotency of these cells. Moreover, TGF-β1 had an inhibitory effect when present at later stages of reprogramming. Therefore, we investigated whether TGF-β1 plays a role in maintaining hiPS cell pluripotency in fully defined culture conditions. TGF-β superfamily members participate in cell fate decisions in ESCs. However, the role of TGF-β in regulating the cell cycle of ESCs is poorly understood. TGF-β/Activin A are essential for the self-renewal of hESCs [22–25], and they function by activating Smad 2, 3 via binding to the Alk4/Activin receptor. Upon activation and dimerization, Smad 2, 3 maintains the pluripotent state through regulation of Nanog transcription [26,27]. Activation of Smad 2, 3 and its downstream targets, such as Nanog, by Activin A/Nodal, and the activation of PI3K/Akt signaling by factors such as IGF-1, heregulin, and FGF-2 are required to maintain pluripotency [26–28]. These two signaling requirements can be identified in all hES cell media formulations described to date. The activity of PI3K/Akt allows Activin A/Smad 2, 3 signaling to promote self-renewal. In the absence of PI3K signaling, Smad 2, 3 collaborates with Wnt pathway effectors to promote differentiation. Recently, TGF-β has been reported to repress activity of the telomerase reverse transcriptase [29]. The role of most members of the TGF-β superfamily in ESCs stemness or differentiation has not yet been investigated so it remains to be established what the specific role of each member of the family is and how it exerts its action under controlled experimental conditions.

In this study, we showed that TGF-β1 increased the expression levels of pluripotency markers such as Oct3/4 and Nanog in dose-dependent manner confirmed by Droplet digital PCR, microarray, and FACS analysis. On the other hand, increasing doses of TGF-β1 suppressed the growth rate of hiPSCs cultured under defined conditions. As with any pleiotropic factor, the effects of TGF-β superfamily members depend on their concentrations well as upon the presence of other factors. Furthermore, in the short term hiPSCs cultured in hESF9 or hESF9T exhibited similar morphologies, but the hiPSCs maintained in hESF9 did not survive beyond 30 passages. This result clearly confirmed that the iPSCs cultured in hESF9 medium absolutely required TGF-β1 to maintain pluripotency. At the same time, when TGF-β1 was present throughout the reprogramming procedure, DPCs tended to overgrow and inhibit or obscure the emergence of iPS colonies. Furthermore, activated p53 and TGF-β1 pathways act as roadblocks for iPSC formation from DPCs [4,24,29]. Decreasing the growth rate of DPCs led to the emergence of iPS cells without DPCs overgrowth. Regulation of TGF-β activity is important for hiPSCs generation and maintenance. Use of a feeder-free defined culture system to generate hiPSCs allowed us to clearly observe the reprogramming process and to begin to analyze the mechanisms involved.

In this study, we showed that iPSCs can be generated from adult DPCs by retroviral transduction of the four transcription factors Oct3/4, Sox2, c-Myc, and Klf-4. Human third molars are discarded as clinical waste and so could be obtained without any further surgical intervention. These teeth are aseptically obtained from the mandible and protected from UV and other damage by surrounding hard tissues. Therefore DPCs are a useful cell source for the generation of iPSCs [30–32]. Clonally expanded DPCs in serum-free medium could be reprogrammed with high iPS generation efficiency. Consequently, the cells are available for iPS generation by other methods using plasmids [33–35], chemicals and proteins [36,37], and microRNAs [38], aiming for the clinical use of the iPS cells in regenerative medicine.

The simplified defined medium described here consists of basal medium and known components that provide a much cleaner background for examining specific signaling pathways in

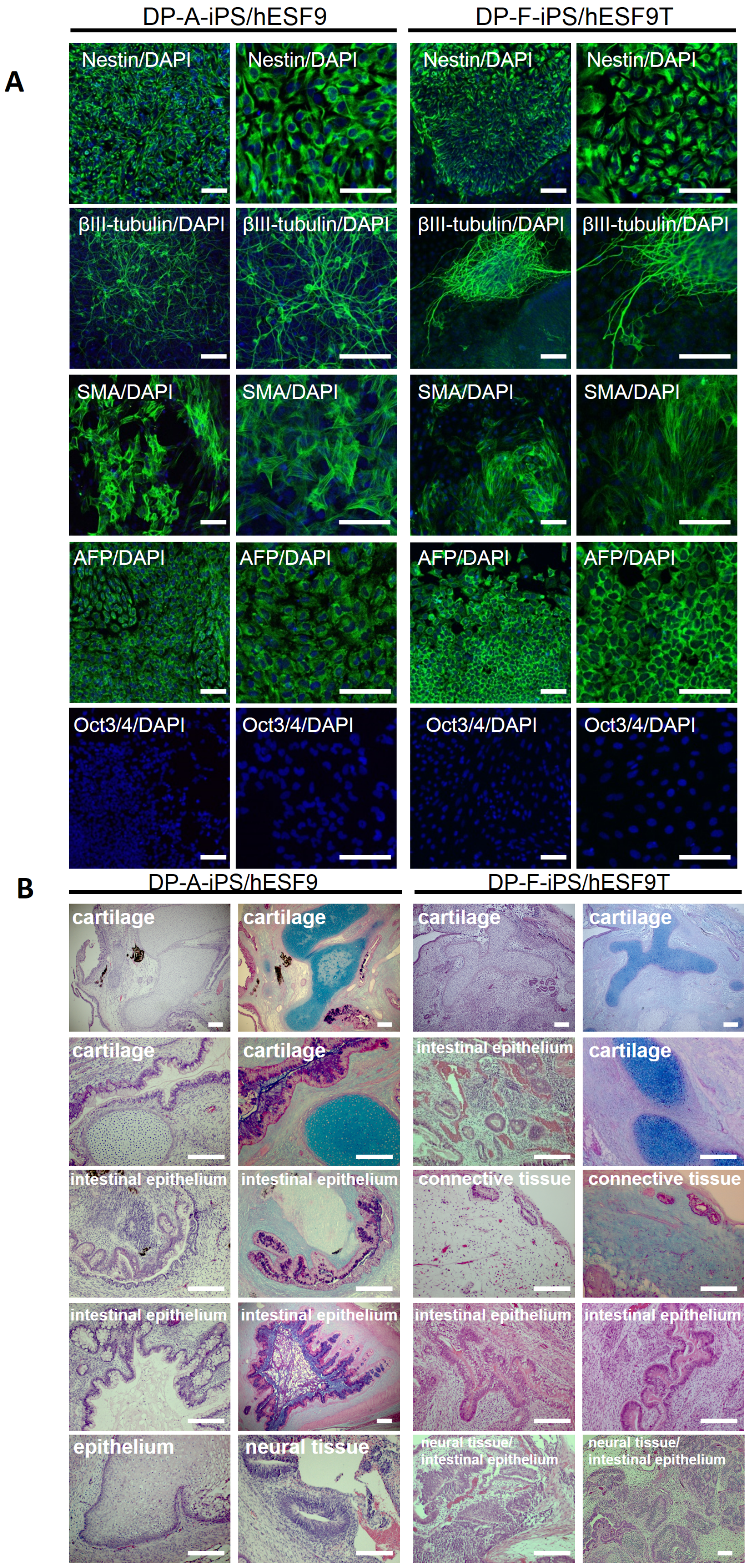
A
DP-A-iPS/hESF9
DP-F-iPS/hESF9T
Nestin/DAPI
βIII-tubulin/DAPI
SMA/DAPI
AFP/DAPI
Oct3/4/DAPI
B
DP-A-iPS/hESF9
DP-F-iPS/hESF9T
cartilage
intestinal epithelium
connective tissue
epithelium
neural tissue
neural tissue/ intestinal epithelium

Figure 6. Embryoid body-mediated differentiation of hiPSCs derived from DPCs in serum-free and feeder-free defined culture conditions and teratoma formation of hiPSCs in the defined culture conditions. A) Differentiation was performed using embryoid body formation, and the differentiated iPSCs (DP-A-iPS/hESF9 or DP-F-iPS/hESF9T) were fixed and reacted with antibodies. Shown were immunocytochemistry of Nestin, βIII-tubulin, α-smooth muscle actin (α-SMA), and α-fetoprotein (AFP). Binding of these antibodies was visualized with Alexa Fluor 488-conjugated secondary antibodies (green). Oct3/4 was also investigated. Binding of these antibodies was visualized with Alexa Fluor® 594-conjugated secondary antibodies (red). Nucleuses were stained with DAPI. (passage 25). Bar indicates 100 μm. B) Teratomas were generated in SCID mice (CB17/Icr-*Prkdc*^{scid}/CrlCrlj) from DP-A-iPS and DP-F-iPS grown under hESF9 or hESF9T-based conditions. Histological analysis with HE staining or Alcian Blue staining demonstrated that teratomas formed from iPS cells cultured in KSR-based (data not shown) or in hESF9T-based conditions contained derivatives of all three germ layers. Left panel shows teratomas from DP-A-iPS-CL1 at passage 22. Right panel shows teratomas from DP-F-iPS-CL14 at passage 6. Scale bars represent 200 μm.
doi:10.1371/journal.pone.0087151.g006

self-renewal, cell death, and cell differentiation, and it supports substantially improved reprogramming efficiencies. Although we have only demonstrated improved efficiencies for viral-based reprogramming, these conditions should be equally useful for other non-integrative reprogramming approaches [33–40]. Finally, since hESF9T medium is defined, it should also help facilitate the transfer of basic research on human pluripotent stem cells to the clinic and useful for understanding disease mechanisms, drug screening, and toxicology.

Conclusions

We have successfully generated hiPSCs from adult human dental pulp cells (DPCs) and maintained them in an undifferentiated state in serum-free defined medium. Furthermore these generated hiPSCs continued to proliferate and retained the properties of self-renewal and pluripotency for a prolonged period of time in the presence of appropriate amount of TGF-β1. As a result, we found TGF-β1 to be an important factor in maintaining pluripotency of hiPSCs. As this simple serum-free adherent monoculture system allows us to elucidate cellular responses to growth factors under defined conditions, these advantages will help to clarify the molecular mechanisms at play in early development.

Supporting Information

Figure S1 Transduction Efficiency of Retroviruses in TIG-3. TIG-3 was introduced with pMXs retroviruses containing the EGFP cDNA. After 4 days, cells were photographed under a fluorescence microscope and analyzed by flow cytometry. The upper panel shows the images of phase contrast and fluorescent microscope. The lower panel shows the result of flow cytometry. Shown are percentages of cells expressing EGFP.
(TIF)

Figure S2 Morphology of transduced TIG-3 on each ECMs in hESF9 medium. A) Upper figures: Twenty days after transduction TIG-3-derived human iPS colony were picked up and sub-cultured on each ECMs. Lower figures: Images of sub-cultured iPS colonies seeded on each ECMs with hESF9 medium for the indicated days at the left. B) Expression of ES cell marker genes in iPSCs derived from TIG-3 cultured on each ECMs with hESF9 medium at day 4. The expression of pluripotency marker genes; Nanog were weakened or disappeared when picked up and sub-cultured on collagen and gelatin. We used primers that only amplified the endogenous genes. #1: hiPSCs generated from TIG-3 on gelatin-coated dish and sub-cultured on gelatin-coated dishes with hESF9 medium at passage 2. #2: hiPSCs generated from TIG-3 on collagen-coated dish and sub-cultured on collagen-coated dishes with hESF9 medium at passage 2. #3: hiPSCs generated from TIG-3 on fibronectin-coated dish and sub-cultured on fibronectin-coated dishes with hESF9 medium at passage 2. Bars indicate 200 μm.
(TIF)

Figure S3 Transduction Efficiency of Retroviruses in Dental Pulp cells. DPCs were introduced with pMXs retroviruses containing the EGFP cDNA. After 4 days, cells were photographed under a fluorescence microscope and analyzed by flow cytometry. The upper panel shows the images of phase contrast and fluorescent microscope. The lower panel shows the result of flow cytometry. Shown are percentages of cells expressing GFP. Transfection efficiency of EGFP was 92.1% in serum-supplemented condition and 89.9% in serum-free culture condition of transfected cells. Bars indicate 200 μm.
(TIF)

Figure S4 hiPS cell generation from DPCs in serum- and feeder-free culture conditions. Images of DPCs (DP-F) plated on collagen-coated dish in RD6F medium. A) Images of DPCs (passage 2) on type I collagen-coated plate with RD6F medium. B) Transduced DPCs were cultured on fibronectin with hESF9 medium or on MEF with KSR-based conditions. After 20 days, iPS colony were picked up and sub-cultured on fibronectin. The reprogramming efficiency was 0.25% with a high success rate. C) ALP staining of iPSCs on fibronectin at 33 days after infection. Bars indicate 200 μm.
(TIF)

Figure S5 Global gene expression analysis of hiPSCs from DPCs. The gene expression of DP-hiPSCs generated in hESF9 and maintained in hESF9T is similar to that of the cells generated and maintained in conventional KSR-based condition or that of Tic (JCRB1331) maintained in conventional KSR-based condition.
(TIF)

Figure S6 karyotype of hiPSC generated in hESF9 and maintained in hESF9T defined culture. A) Growth curve of hiPSCs. Shown were averages. Growth curves for the hiPSC (DP-F-iPS-CL16) cultured under hESF9T at passage 21, 22, 23 and 24 were seeded in a 24-well plate coated with fibronectin and the cell numbers were counted every 24 h. The values are the mean± SEM (n = 4). Population doubling time: 16.6±0.843 h. B) Karyotype analysis of DP-F-iPS-CL14 cell at passage 20 maintained in hESF9T conditions. Normal diploid 46, XX karyotype.
(TIF)

Table S1 Composition of medium used for serum-free culture. The composition of the basal medium RD is described in Sato, JD et al., 1987[11]. hESF9 medium is described in Furue et al., 2008 [5].
(TIF)

Table S2 Primers used in this study listed.
(TIF)

Table S3 STR analyses of DP-derived iPSCs.
(TIF)

Acknowledgments

We thank Dr. J. Denry Sato for editorial assistance. The authors are grateful to Dr. Miho K. Furue at NIBIO for valuable suggestions. We are also grateful to Ms. Michiko Nii, Atsuko Hamada and Eri Akagi at Hiroshima University for karyotype analysis of the hiPSCs.

Author Contributions

Conceived and designed the experiments: SY AS TO. Performed the experiments: SY YT HM. Analyzed the data: SY AS TO. Contributed reagents/materials/analysis tools: AS HT TO. Wrote the paper: SY TO.

References

1. Takahashi K, Tanabe K, Ohnuki M, Narita M, Ichisaka T, et al. (2007) Induction of pluripotent stem cells from adult human fibroblasts by defined factors. Cell 131: 861–872.
2. Yu J, Vodyanik MA, Smuga-Otto K, Antosiewicz-Bourget J, Frane JL, et al. (2007) Induced pluripotent stem cell lines derived from human somatic cells. Science 318: 1917–1920.
3. Hayashi Y, Chan T, Warashima M, Fukuda M, Ariizumi T, et al. (2010) Reduction of N-Glycolylneuraminic Acid in Human Induced Pluripotent Stem Cells Generation or Cultured under Feeder-and Serum-Free Defined Condition. PLoS One 5: e14099.
4. Chen G, Gulbranson DR, Hou Z, Bolin JM, Ruotti V, et al. (2011) Chemically defined conditions for human iPSC derivation and culture. Nat Methods 8: 424–429.
5. Furue MK, Na J, Jackson JP, Okamoto T, Jones M, et al. (2008) Heparin promotes the growth of human embryonic stem cells in a defined serum-free medium. Proc Natl Acad Sci USA 105: 13409–13414.
6. Morita S, Kojima T, Kitamura T (2000) Plat-E: an efficient and stable system for transient packaging of retroviruses. Gene Ther 7:1063–1066.
7. Matsuo M, Kaji K, Utakoji T, Hosoda K (1982) Ploidy of human embryonic fibroblasts during in vitro aging. J Gerontol 37: 33–37.
8. Takahashi K, Okita K, Nakagawa M, Yamanaka S (2007) Induction of pluripotent stem cells from fibroblast cultures. Nat Protoc 2:3081–3089.
9. Draper JS, Moore HD, Ruban LN, Gokhale PJ, Andrews PW (2004) Culture and characterization of human embryonic stem cells. Stem Cells Dev 13: 325–36.
10. Park IH, Zhao R, West JA, Yabuuchi A, Huo H, et al. (2008) Reprogramming of human somatic cells to pluripotency with defined factors. Nature 451:141–146.
11. Sato JD, Kawamoto T, Okamoto T (1987) Cholesterol requirement of P3-X63-Ag8 and X63-Ag8.653 mouse myeloma cells for growth in vitro. J Exp Med 165:1761–1766.
12. Myoken Y, Okamoto T, Osaki T, Yabumoto M, Sato GH, et al. (1989) An alternative method for the isolation of NS-1 hybridomas using cholesterol auxotrophy of NS-1 mouse myeloma cells. In Vitro Cell Dev Biol 25: 477–480.
13. Furue M, Okamoto T, Hayashi Y, Okochi H, Fujimoto M, et al. (2005) Leukemia inhibitory factor as anti-apoptotic mitogen for pluripotent mouse embryonic stem cells in a serum-free medium without feeder cells. In Vitro Cell Dev Biol Anim 41: 19–28.
14. Rajala K, Hakala H, Panula S, Aivio S, Pihlajamäki H, et al. (2007) Testing of nine different xenofree culture media for human embryonic stem cell cultures. Hum Reprod 22:1231–1238.
15. Gu J, Fujibayashi A, Yamada KM, Sekiguchi K (2002) Laminin-10/11 and fibronectin differentially prevent apoptosis induced by serum removal via phosphatidylinositol 3-kinase/Akt- and MEK1/ERK-dependent pathways. J Biol Chem 277:19922–19928.
16. Braam SR, Zeinstra L, Litjens S, Ward-van Oostwaard D, van den Brink S, et al. (2008) Recombinant vitronectin is a functionally defined substrate that supports human embryonic stem cell self-renewal via alphavbeta5 integrin. Stem Cells 26: 2257–2265.
17. Prowse AB, Doran MR, Cooper-White JJ, Chong F, Munro TP, et al. (2010) Long term culture of human embryonic stem cells on recombinant vitronectin in ascorbate free media. Biomaterials 31: 8281–8288.
18. Lu J, Hou R, Booth CJ, Yang SH, Snyder M (2006) Defined culture conditions of human embryonic stem cells. Proc. Natl Acad. Sci. USA 103: 5688–5693.
19. Rodin S, Domogatskaya A, Ström S, Hansson EM, Chien KR, et al. (2010) Long-term self-renewal of human pluripotent stem cells on human pluripotent stem cells on human recombinant laminin-511. Nat biotechnol 28: 611–615.
20. Miyazaki T, Futaki S, Suemori H, Taniguchi Y, Yamada M, et al. (2012) Laminin E8 fragments support efficient adhesion and expansion of dissociated human pluripotent stem cells. Nat Commun 3:1236.
21. Bendall SC, Stewart MH, Menendez P, George D, Vijayaragavan K, et al. (2007) IGF and FGF cooperatively establish the regulatory stem cell niche of pluripotent human cells in vitro. Nature 488:1015–1021.
22. Kunath T, Saba-El-Leil MK, Almousailleakh M, Wray J, Meloche S, et al. (2007) FGF stimulation of the Erk1/2 signaling cascade triggers transition of pluripotent embryonic stem cells from self-renewal to lineage commitment. Development 134:2895–2902.
23. Beattie GM, Lopez AD, Bucay N, Hinton A, Firpo MT, et al. (2005) Activin A maintains pluripotency of human embryonic stem cells in the absence of feeder layers. Stem Cells 23:489–495.
24. James D, Levine AJ, Besser D, Hemmati-Brivanlou A (2005) TGFbeta/activin/nodal signaling is necessary for the maintenance of pluripotency in human embryonic stem cells Development 132:1273–1282.
25. Xiao L, Yuan X, Sharkis SJ (2006) Activin A maintains self-renewal and regulates fibroblast growth factor, Wnt, and bone morphogenic protein pathways in human embryonic stem cells. Stem Cells 24:1476–1486.
26. Xu RH, Sampsell-Barron TL, Gu F, Root S, Peck RM, et al. (2008) NANOG is a direct target of TGF beta/activin-mediated SMAD signaling in human ESCs. Cell Stem Cell 3:196–206.
27. Vallier L, Mendjan S, Brown S, Chng Z, Teo A, et al. (2009) Activin/Nodal signaling maintains pluripotency by controlling Nanog expression. Development 136: 1339–1349.
28. Singh AM, Reynolds D, Cliff T, Ohtsuka S, Mattheyses AL, et al. (2012) Signaling network crosstalk in human pluripotent cells: a Smad2/3-regulated switch that controls the balance between self-renewal and differentiation. Cell Stem Cell 10:312–326.
29. Li H, Xu D, Li J, Berndt MC, Liu JP (2006) Transforming growth factor beta suppresses human telomerase reverse transcriptase by Smad3 interactions with C-Myc and hTERT gene. J Biol Chem 35:25588–25600.
30. Tamaoki N, Takahashi K, Tanaka T, Ichisaka T, Aoki H, et al. (2010) Dental pulp cells for induced pluripotent stem cell banking. J Dent Res 89:773-778.
31. Oda Y, Yoshimura Y, Ohnishi H, Tadokoro M, Katsube Y, et al. (2010) Induction of pluripotent stem cells from human third molar mesenchymal stromal cells. J Biol Chem 285: 29270–29278.
32. Yan X, Qin H, Qu C, Tuan RS, Shi S, et al. (2010) iPS cells reprogrammed from human mesenchymal-like stem/progenitor cells of dental tissue origin. Stem Cells Dev 19:469–480.
33. Okita K, Nakagawa M, Hyenjong H, Ichisaka T, Yamanaka S (2008) Generation of mouse induced pluripotent stem cells without viral vectors. Science 322:949–953.
34. Stadtfeld M, Nagaya M, Utikal J, Weir G, Hochedlinger K (2008) Induced pluripotent stem cells generated without viral integration. Science 322:945–949.
35. Yu J, Hu K, Smuga-Otto K, Tian S, Stewart R, et al. (2009) Human induced pluripotent stem cells free of vector and transgene sequences. Science 324:797–801.
36. Zhu S, Li W, Zhou H, Wei W, Ambasudhan R, et al. (2010) Reprogramming of human primary somatic cells by OCT4 and chemical compounds. Cell Stem Cell 7:651–655.
37. Kim D, Kim CH, Moon JI, Chung YG, Chang MY, et al. (2009) Generation of human induced pluripotent stem cells by direct delivery of reprogramming proteins. Cell Stem Cell 4:472–476.
38. Miyoshi N, Ishii H, Nagano H, Haraguchi N, Dewi DL, et al. (2011) Reprogramming of mouse and human cells to pluripotency using mature microRNAs. Cell Stem Cell 8:633–638.
39. Nakagawa M, Koyanagi M, Tanabe K, Takahashi K, Ichisaka T, et al. (2008) Generation of Induced Pluripotent Stem Cells without Myc from mouse and human fibroblasts. Nat Biotechnol 26:101–106.
40. Nakagawa M, Takizawa N, Narita M, Ichisaka T, Yamanaka S (2010) Promotion of direct reprogramming by transfection-deficient myc. Proc Natl Acad Sci U S A 107:14152–14157.
